# Transcriptomic profiling of thyroid eye disease orbital fat demonstrates differences in adipogenicity and IGF-1R pathway

**DOI:** 10.1172/jci.insight.182352

**Published:** 2024-12-20

**Authors:** Dong Won Kim, Soohyun Kim, Jeong Han, Karan Belday, Emily Li, Nicholas Mahoney, Seth Blackshaw, Fatemeh Rajaii

**Affiliations:** 1 Danish Research Institute of Translational Neuroscience (DANDRITE), Nordic EMBL Partnership for Molecular Medicine, and; 2 Department of Biomedicine, Aarhus University, Aarhus, Denmark.; 3 Department of Ophthalmology, Wilmer Eye Institute, Johns Hopkins University School of Medicine, Baltimore, Maryland, USA.; 4 Baylor College of Medicine, Houston, Texas, USA.; 5 Department of Neurology,; 6 Institute for Cell Engineering, and; 7 Kavli Neuroscience Discovery Institute, Johns Hopkins University School of Medicine, Baltimore, Maryland, USA.

**Keywords:** Inflammation, Ophthalmology, Adipose tissue, Thyroid disease

## Abstract

Despite recent advances in the treatment of thyroid eye disease thyroid-related eye disease (TED), marked gaps remain in our understanding of the underlying molecular mechanisms, particularly concerning the insulin-like growth factor-1 receptor (IGF-1R) pathway. To dissect the pathophysiology of TED, we used single-nucleus RNA-Seq to analyze orbital fat specimens from both patients with TED and matched individuals acting as controls. The analysis demonstrated a marked increase in the proportion of fibroblasts transitioning to adipogenesis in the orbital fat of patients with TED compared with that in control patients. This was associated with diverse alterations in immune cell composition. Significant alterations in the IGF-1R signaling pathway were noted between TED specimens and those from control patients, indicating a potential pathological mechanism driven by IGF-1R signaling abnormalities. Additionally, our data showed that linsitinib, a small-molecule inhibitor of IGF-1R, effectively reduced adipogenesis in TED orbital fibroblasts in vitro, suggesting its potential utility as a therapeutic agent. Our findings reveal that, beyond immune dysfunction, abnormal IGF-1R signaling leading to enhanced adipogenesis is a crucial pathogenic mechanism in TED.

## Abstract 2

We utilized snRNA-Seq to explore the cellular landscape of orbital fat in TED, providing in vivo evidence of increased adipogenesis and IGF-1 pathway dysfunction.

## Introduction

Autoimmune thyroid disease is the most prevalent organ-specific autoimmune disease, affecting 2%–5% of the global population. Approximately 25%–40% of patients with autoimmune thyroid disease eventually develop thyroid-related eye disease (TED), a condition that can lead to disfigurement and vision loss (1–4). Although the pathophysiology of TED is not entirely understood, it is thought that an immune response targeting autoantigens present on thyrocytes and orbital fibroblasts, the thyroid-stimulating hormone receptor (TSHR) and/or the insulin-like growth factor-1 receptor (IGF-1R), induces a cascade of inflammatory cytokines (5–15). This process increases orbital soft tissue volume through mechanisms such as glycosaminoglycan production, fibrosis, and adipogenesis, all of which have been demonstrated in vitro (12, 13, 15). The expansion of orbital soft tissues, responsible for proptosis, diplopia, pain, and vision loss in TED, is thought to be the final step of inflammatory cascades that result in fibrosis and adipogenesis.

IGF-1 enhances the expression of TSHR in orbital fibroblasts, and treatment with the monoclonal anti–IGF-1R antibody teprotumumab reduces TSHR and IGF-1R expression (16). Moreover, teprotumumab blocks the induction of inflammatory cytokines IL-6 and IL-8 in orbital fibroblasts (17). Single-nucleus RNA-Seq (snRNA-Seq) has identified that during the early stages of adipogenesis, orbital fibroblasts upregulate the expression of both IGF-1 and IGF-1R in vitro (18).

In this study, we aimed to demonstrate the role of adipogenesis in TED in vivo. We utilized snRNA-Seq to compare gene expression profiles in retrobulbar fat from individuals acting as controls undergoing orbital surgery and compared it with those of patients with TED undergoing orbital decompression. Our findings not only confirmed an increase in adipocytes but also highlighted an increased presence of fibroblasts undergoing adipogenesis in the orbital fat of patients with TED. Furthermore, we observed disruptions in the IGF pathway, including IGF-1R signaling in both the orbital fibroblasts and fat of patients with TED. To investigate the function of the IGF-1R signaling pathway in orbital adipogenesis (18), we employed linsitinib, a small-molecule antagonist of IGF-1R. Our in vitro studies demonstrated that blocking IGF1-R signaling reduces adipogenesis in TED orbital fibroblasts, providing what we believe to be new insights into the pathophysiological mechanisms of TED and identifying potential therapeutic targets.

## Results

### TED human orbital tissue shows increased adipocytes.

**Figure 1:**
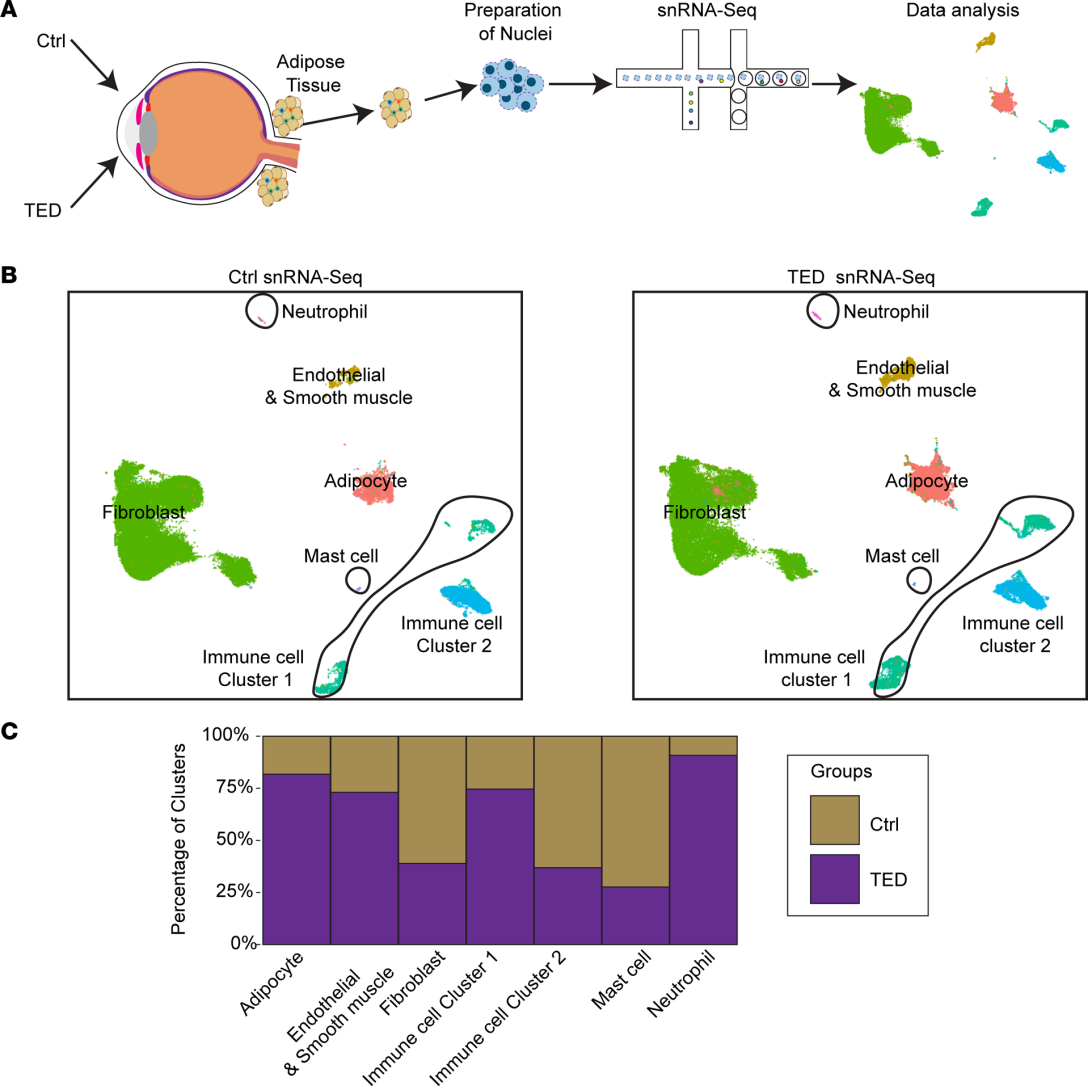
Overview of the experimental approach and cell-type distribution in the control and TED groups. (**A**) Schematic of the experimental workflow. (**B**) UMAP plots illustrating the distribution of cell types in the control (left) and TED (right) groups. (**C**) Bar graphs comparing the distribution of cell clusters between the control and TED groups identified in snRNA-Seq.

To delineate changes in the cellular composition within orbital tissue associated with TED, we utilized snRNA-Seq to analyze samples from patients diagnosed with TED in comparison with samples from control patients without TED pathology (Figure 1A and Supplemental Table 1; supplemental material available online with this article; https://doi.org/10.1172/jci.insight.182352DS1). The patients with TED ranged in age from 22 to over 70 years, while control patients were mostly over 70 years of age. Smoking history was limited, with only 1 individual acting as a control reporting smoking more than 10 years ago and another reporting rare use (Supplemental Table 1). Our analysis identified 4 primary cell populations present in both groups: fibroblasts, adipocytes, endothelial cells, and a diverse array of immune cells (Figure 1B). Fibroblasts were characterized by the expression of genes such as *DCN*, *CNTN4*, and *ADAMTSL1* (Supplemental Table 2). Adipocytes were identified through the expression of *PDE3B*, *LPL*, and *PLIN1* (Supplemental Table 2). Endothelial and smooth cells exhibited *VWF* expression, and the immune cell population comprised multiple subsets, including neutrophils, mast cells, and cells indicative of either innate (immune cell cluster 2, *F13A1* and *MRC1*) or adaptive (immune cell cluster 1, *PTPRC* and *RIPOR2*) responses (Supplemental Table 2).

Notably, the group of patients with TED exhibited a substantial increase in adipocyte numbers compared with the control group (Figure 1C and Supplemental Figure 1), consistent with the adipogenic shift previously documented in TED pathology (18). Increases in *IGF1*, *IGF1R*, and *TSHR* in TED fibroblasts compared with the controls were observed (Supplemental Figure 2), aligning with previous studies (19). While increases in *IGF1* and *TSHR* were observed in TED adipocytes compared with controls, *IGF1R* levels did not differ between the groups (Supplemental Figure 2). *IGF2* expression was absent in both groups (Supplemental Figure 2).

Furthermore, adipogenic markers such as *PDE3B* and *PLIN1* were detected in TED fibroblasts but not in controls (Supplemental Figure 3), suggesting an ongoing adipogenic transition within TED fibroblasts. Additionally, our snRNA-Seq data highlighted an increased presence of immune cells facilitating adaptive responses in TED samples, contrasting with a diminished representation of cells associated with innate immunity (Figure 1C). These observations not only validate known histopathological features in TED but also reveal insights into the disease’s immune landscape.

### Exploring variations in immune cell distribution between the control and TED groups.

**Figure 2:**
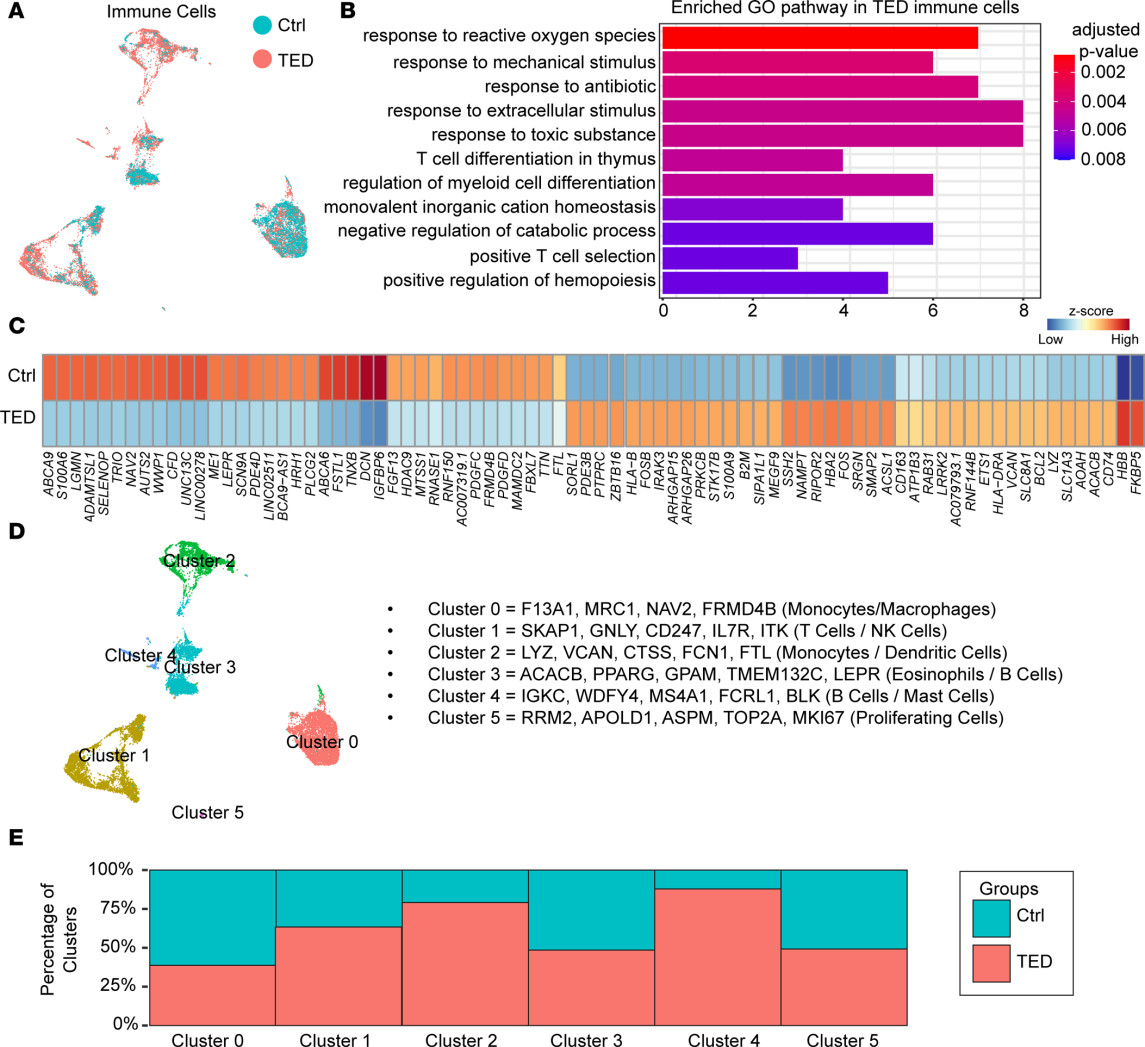
Immune cell analysis and pathway enrichment in the control and TED groups. (**A**) UMAP plot of immune cells in the control and TED groups. (**B**) GO pathway analysis of genes enriched in TED immune cells. (**C**) Heatmap displaying the expression of control- or TED-enriched genes in immune cells. (**D**) UMAP plot showing identified subclusters of immune cells across all samples. (**E**) Bar graphs comparing the distribution of immune cell clusters between the control and TED groups identified in snRNA-Seq.

To further explore the variations in immune cell distribution between the control and TED groups, we focused our analysis on immune cell clusters 1 and 2. These clusters were characterized by gene expressions indicative of adaptive (cluster 1) and innate (cluster 2) immune responses (Supplemental Table 2). A Gene Ontology (GO) pathway analysis of TED immune cells (Figure 2, A and B, and Supplemental Table 3), revealed pathways such as “T cell differentiation in thymus” and “positive T cell selection.” This analysis also uncovered heightened expressions of *CD74*, *LYZ*, *HLA-DBA*, *CD163*, and *IRAK3* in TED immune cells (Figure 2C and Supplemental Table 3) — genes generally enriched in adaptive immune cells. Such findings suggest a pronounced shift toward an adaptive immune response in TED, consistent with autoimmune etiology (20, 21).

Further subdivision of immune cells into 6 distinct clusters (Figure 2D) revealed diverse contributions from each cluster between the groups (Figure 2E). Although the specific immune cell subtypes within each cluster remained unidentified (Supplemental Table 4), the markers noted suggest a composition enriched in monocytes/macrophages (clusters 0 and 2), NK cells and T cells (cluster 1), dendritic cells (cluster 2), and B cells (clusters 3 and 4). Cluster 1 cells from patients with TED showed elevated expression of CD4^+^ T cell markers, aligning with findings from a previous study that identified CD4^+^ T cells associated with hyperthyroidism and TED (22). These markers include *PRF1*, *GZMA*, *GNLY*, and *GZMH* (Supplemental Figure 4).

### Differential expression of the IGF signaling pathway among TED orbital fibroblasts.

**Figure 3:**
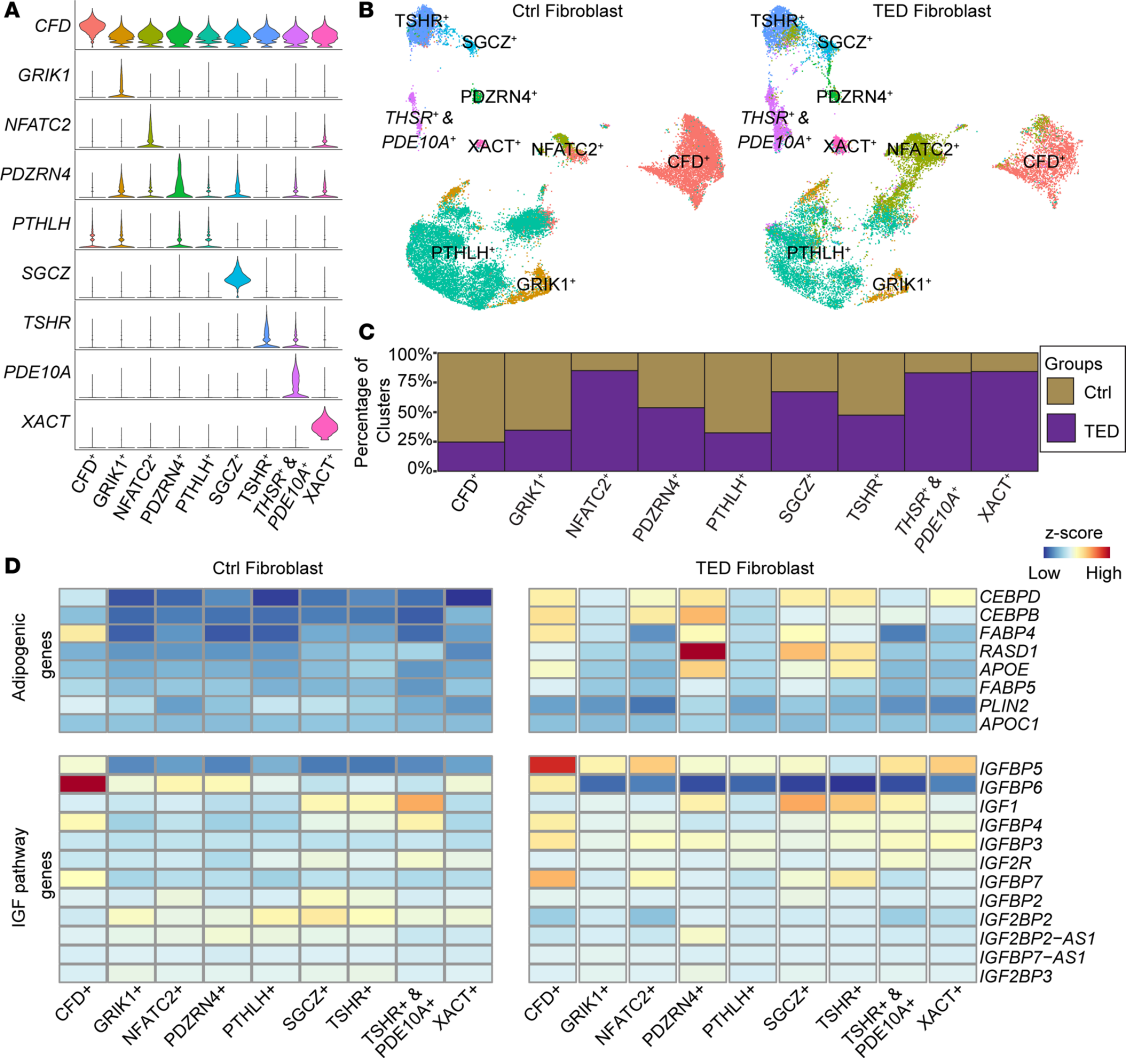
Analysis of orbital fibroblast subtypes and adipogenic gene expression in the control and TED groups. (**A**) Violin plots showing genes enriched in specific clusters across orbital fibroblast subtypes in all samples. (**B**) UMAP plot showing the distribution of orbital fibroblast subclusters in control (left) and TED (right) samples. (**C**) Bar graphs comparing the distribution of orbital fibroblast subclusters between the control and TED groups. (**D**) Heatmap displaying the expression of adipogenic and IGF-related genes across groups.

Further investigation of orbital fibroblast populations from TED and control samples identified multiple subclusters of orbital fibroblasts, each distinguished by unique molecular markers (Figure 3, A and B, and Supplemental Table 5). A GO pathway analysis of genes enriched within these clusters identified varied biological pathways (Supplemental Figure 5). While some clusters showed no unique pathway alterations, the clusters predominantly found in TED samples exhibited distinctive pathway modifications (Supplemental Figure 5). Specifically, clusters labeled as “NFATC2,” “TSHR & PDE10A,” and “XACT” were markedly prevalent in TED samples (Figure 3C), and these clusters displayed changes in pathways related to “response to steroid hormone” and “cellular response.” These findings suggest biological shifts associated with TED or effects of therapeutic interventions like radiotherapy or drug treatments on mitigating TED symptoms (Supplemental Figure 5).

Despite the variability in molecular markers across fibroblast subclusters, a commonality in the expression of adipocyte-enriched genes (18) was observed between TED and control groups (Supplemental Figure 6). TED orbital fibroblasts, irrespective of the cluster, demonstrated higher levels of adipocyte-enriched genes compared with controls (Supplemental Figure 6). Additionally, an examination of genes known to regulate adipogenesis, such as *CEBPB* and *CEBPD* (23), *FABP4* (24), *FABP5* (25), *PLIN2* (26), *APOC1* (27), *APOE* (28), and *RASD1* (29), revealed a comprehensive upregulation in adipogenic genes, notably *CEBPB* and *CEBPD* in all TED-associated orbital fibroblast subclusters (Figure 3D and Supplemental Table 6). This suggests an inherent adipogenic predisposition within TED fibroblasts. However, other markers like *APOE*, *RASD1*, and *FABP4* were selectively upregulated in specific TED fibroblast subclusters (Figure 3D and Supplemental Table 6). Interestingly, the presence of fibroblast subclusters with a higher representation in TED samples did not directly correlate with increased adipogenic gene expression, and no differences were found in the expression of *FABP5, PLIN2,* and *APOC1* between the groups.

Our study also identified disparities in the expression of genes related to the IGF signaling pathway across the fibroblast subclusters. Notably, TED orbital fibroblasts exhibited a significant reduction in *IGFBP6* expression alongside an increase in *IGFBP5* relative to controls (Figure 3D and Supplemental Table 6). This differential expression indicates a distinctive modulation of the IGF signaling pathway in TED fibroblasts, suggesting a unique pathophysiological mechanism at play.

### Enhanced adipogenesis in TED adipocytes.

**Figure 4:**
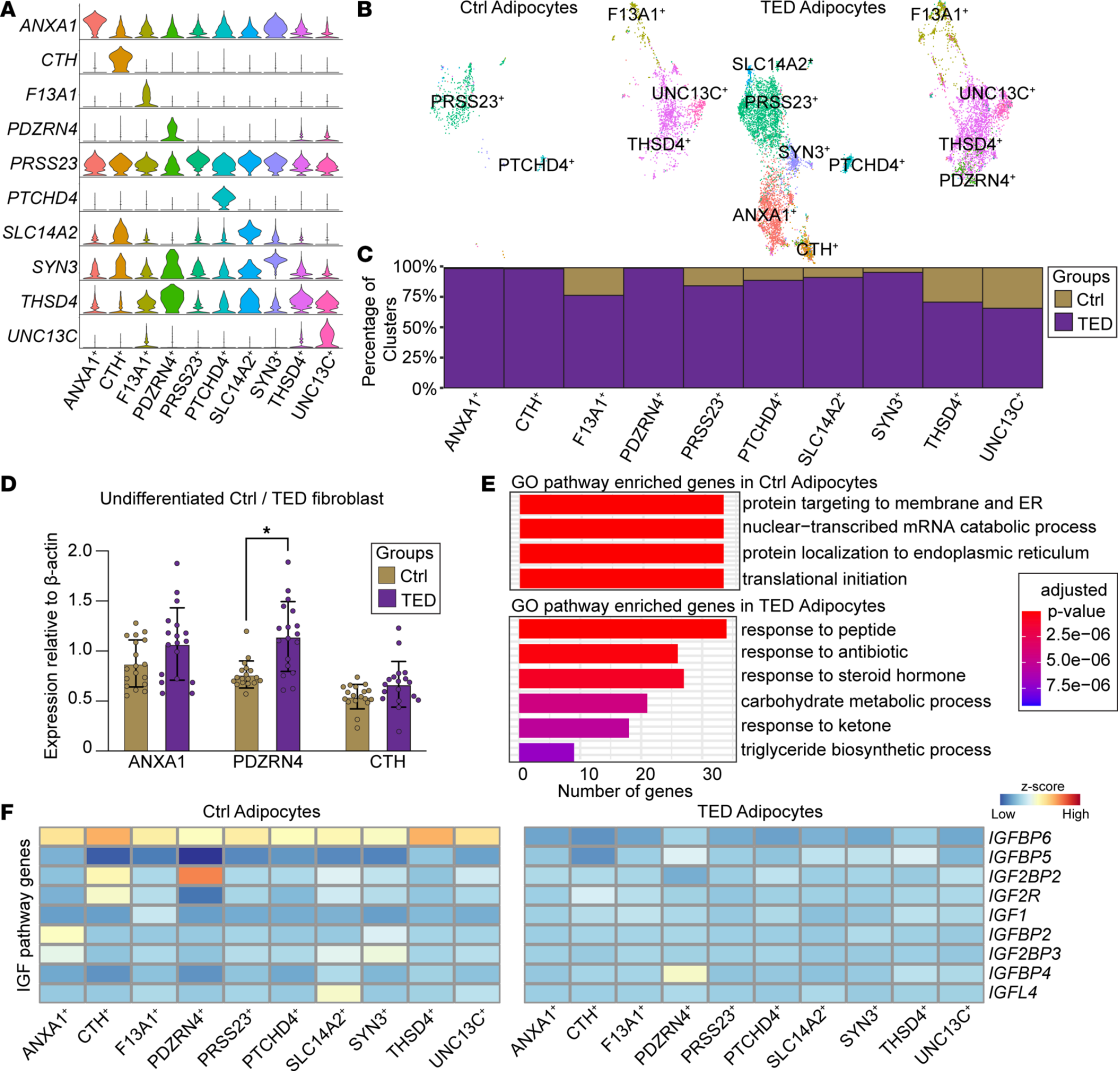
Characterization of orbital adipocyte subtypes and IGF-related gene expression. (**A**) Violin plots showing genes enriched in specific clusters across orbital adipocyte subtypes in all samples. (**B**) UMAP plot showing the distribution of orbital adipocyte subclusters in control (left) and TED (right) groups. (**C**) Bar graphs comparing the distribution of orbital adipocyte subclusters between the control and TED groups. (**D**) Bar graphs showing the expression of *ANXA1*, *PDZRN4*, and *CTH* in undifferentiated control and TED fibroblasts. *n* = 3 cell lines each, performed in triplicate.*P < 0.001 by unpaired *t* test. (**E**) GO pathway analysis of genes enriched in control (left) and TED adipocytes (right). (**F**) Heatmap displaying the expression of IGF-related genes across groups.

In our analysis of adipocytes derived from both TED and control groups, we identified multiple subclusters within each group, each defined by distinct molecular markers (Figure 4A and Supplemental Table 7). Notably, the TED group exhibited a substantially increased number of adipocytes compared with that in the control group (Figure 4B). Additionally, unique adipocyte subclusters named “ANXA1,” “CTH,” and “PDZRN4” were predominantly found in TED samples (Figure 4C). A GO pathway analysis of these subclusters, particularly the CTH subcluster, indicated a distinct shift toward “fat cell differentiation” (Supplemental Figure 7), suggesting that these subclusters may represent recently differentiated adipocytes. To further validate the distinct adipocyte populations identified through snRNA-Seq, Western blot analysis was performed to validate the expression of ANXA1, PDZRN4, and CTH in isolated, undifferentiated orbital fibroblasts from both patients with TED and healthy control patients. PDZRN4 expression was significantly higher in TED fibroblasts compared with control fibroblasts (*n* = 3, *P* < 0.001) (Figure 4D). Additionally, ANXA1 and CTH exhibited a trend toward higher expression in TED fibroblasts, though these differences did not reach statistical significance.

Further comparative GO pathway analysis across all subclusters of control and TED adipocytes revealed notable differences (Figure 4E and Supplemental Figure 7). TED adipocytes were enriched in pathways associated with “response to peptide,” “response to steroid hormone/glucocorticoid,” and “proliferation.” These findings suggest a complex interaction of differentiation and proliferation processes contributing to the increased adipocyte population observed in TED (Figure 4E).

A detailed examination of gene expression within the IGF pathway between the two cohorts further uncovered differences. Despite variability within subclusters, TED adipocytes consistently displayed a decrease in *IGFBP6* expression across all subclusters compared with controls (Figure 4F and Supplemental Table 8), alongside an increase in *IGFBP5* expression (Supplemental Table 8). Additionally, reductions in *IGFL4* and *IGFBP2* expression were observed in TED adipocytes, in contrast to the mixed expression patterns of IGF pathway genes seen in orbital fibroblasts. This pattern indicates a more pronounced dampening of IGF signaling in TED adipocytes.

### Adipogenesis trajectory of TED orbital fibroblasts and adipocytes.

To investigate the adipogenic differentiation of orbital fibroblasts in TED, we conducted an integrated analysis of TED orbital fibroblasts with adipocytes. Due to the limited number of adipocytes in control samples, this analysis was specifically focused on identifying changes within the TED cohort.

**Figure 5:**
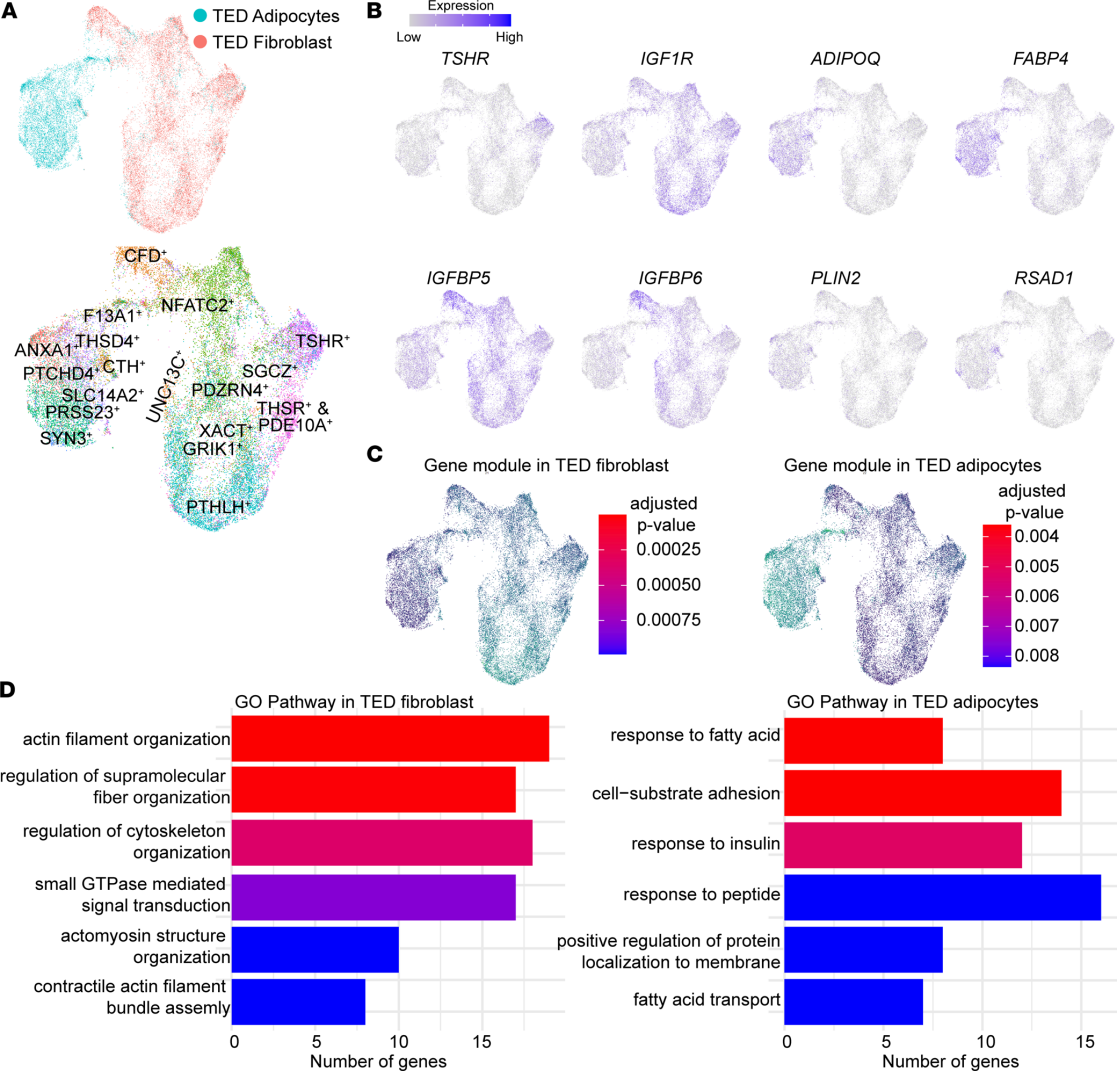
Combined analysis of orbital fibroblasts and adipocytes in TED groups and pathway analysis. (**A**) UMAP plot showing merged orbital fibroblasts and adipocytes in the TED group (top) and previously identified (see Figure 3B and Figure 4B) clusters (bottom). (**B**) UMAP plots showing the expression of cluster-specific/enriched genes. (**C**) Gene modules in TED fibroblast (left) and TED adipocytes (right). (**D**) GO pathway analysis of gene modules in TED fibroblasts (left) and TED adipocytes (right).

Our initial objective was to map a direct adipogenic pathway from specific fibroblast subclusters to differentiated adipocytes. Although the “NFATC2” fibroblast cluster in TED exhibited a closer association with differentiated adipocytes compared with the “CFD” cluster, a definitive adipogenic lineage from any particular fibroblast subcluster to adipocytes could not be conclusively established (Figure 5A).

We observed marked downregulation of *TSHR* in adipocytes compared with orbital fibroblasts (Figure 5B), accompanied by a consistent decrease in the expression of *IGF1R, IGFBP5*, and *IGFBP6* (Figure 5B). Conversely, adipocytes demonstrated an upregulation of adipogenic markers such as *ADIPOQ*, *FABP4, PLIN2,* and *RASD1* (Figure 4B), indicating successful adipogenesis.

A GO pathway analysis delineated distinct biological processes between the two cell types (Figure 5C). TED orbital fibroblasts were predominantly involved in pathways associated with “cytoskeleton organization/assembly” (Figure 5D). In contrast, TED adipocytes were linked to “response to fatty acid” and “insulin response” pathways (Figure 5D), underscoring the substantial role of insulin in adipogenic differentiation from fibroblast to adipocytes (18). These observations further reinforce the importance of the IGF pathway in the adipogenic process in TED, aligning with findings from earlier in vitro studies on patient-derived human orbital fibroblast cell lines (18).

### Linsitinib treatment reduces adipogenesis in TED fibroblasts.

The observed decrease in IGF-1 pathway gene expression in differentiated adipocytes relative to orbital fibroblasts aligns with previous research that IGF-1 signaling initially surges during the early stage of adipogenesis in vitro and then declines as adipocytes mature. This finding prompted us to investigate the effects of manipulating the IGF-1 pathway on adipogenesis in orbital fibroblasts. We utilized linsitinib, a small-molecule IGF-1R antagonist, which diminishes immune infiltration and fibrosis in orbit in TED animal models (30). Currently, linsitinib is undergoing phase II clinical trials as a potential oral therapy for TED.

**Figure 6:**
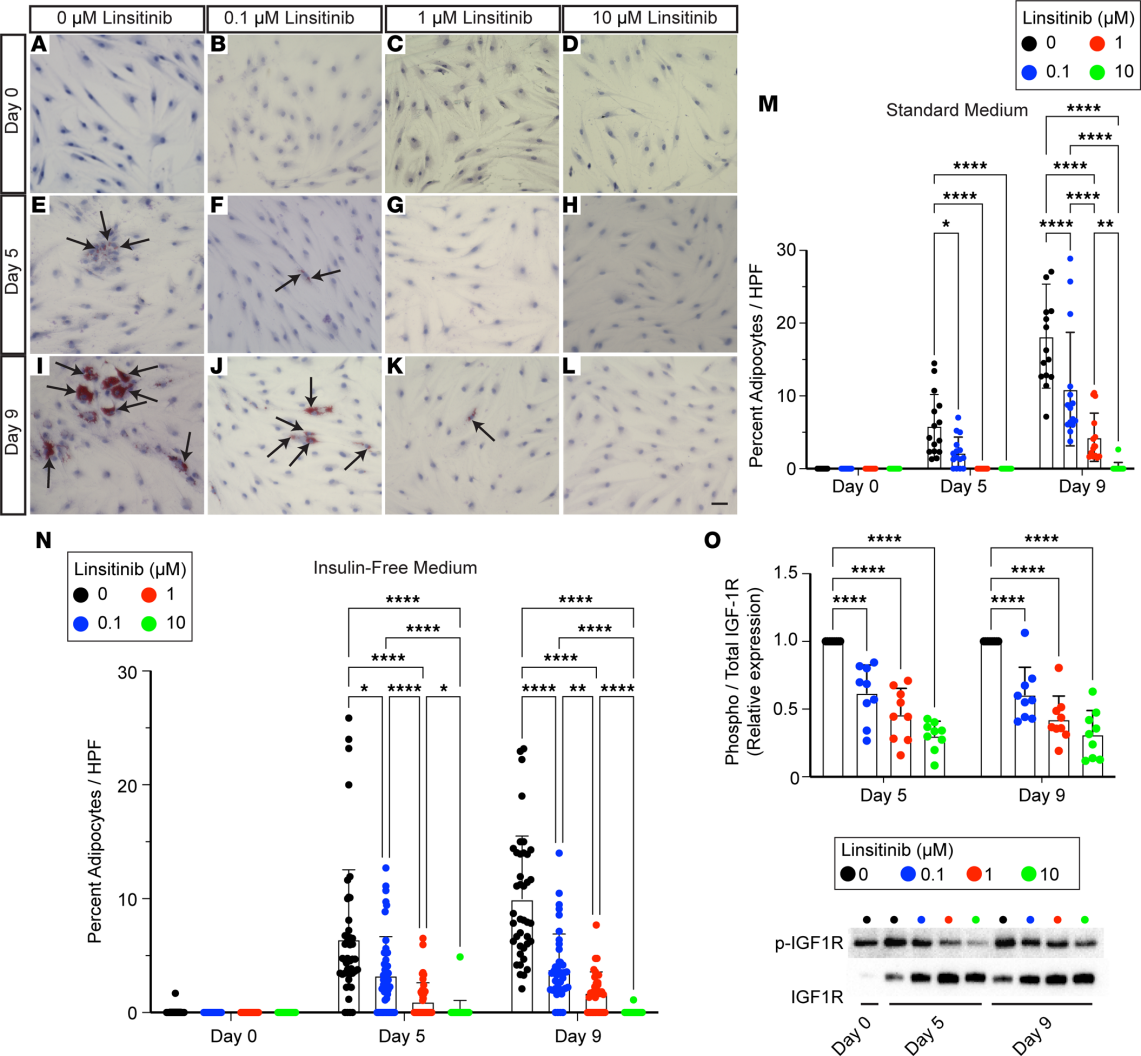
Effect of linsitinib on adipogenesis and IGF1R expression in orbital fibroblasts. (**A**–**L**) Oil Red O staining of orbital fibroblasts treated with adipogenic media (**A**, **E**, and **I**) and 0.1 μM (**B**, **F**, and **J**), 1 μM (**C**, **G**, and **K**), or 10 μM (**D**, **H**, and **L**) linsitinib on days 0 (**A**–**D**), 5 (**E**–**H**), and 9 (**I**–**L**) after treatment. Arrows indicate Oil Red O^+^ adipocytes. Scale bar: 50 μm. (**M**) Bar plot showing the percentage of adipocytes after treatment with standard adipogenic medium and 0, 0.1, 1, or 10 μM linsitinib on days 0, 5, and 9. *P < 0.05; **P < 0.01; ****P < 0.0001 by Tukey’s multiple comparison test. (**N**) Bar plot showing the percentage of adipocytes after treatment with insulin-free adipogenic medium and 0, 0.1, 1, or 10 μM linsitinib on days 0, 5, and 9. *P < 0.05; **P < 0.01; ****P < 0.0001 by Tukey’s multiple comparison test. (**O**) Bar plot (top) and blots (bottom) showing the relative expression between phospho-IGF1R and total IGF1R after treatment with insulin-free adipogenic medium and 0, 0.1, 1, or 10 μM linsitinib on days 5 and 9. ****P < 0.0001 by Šidák’s multiple comparison test.

In our established in vitro model using patient-derived human orbital fibroblast cell lines (18), we administered linsitinib in a dose-dependent manner (Figure 6, A–L). Consistent with prior observations, adipocyte differentiation was evident by day 5 in vitro, as indicated by Oil Red O^+^ adipocytes (Figure 6E). A notable dose-dependent reduction in the proportion of adipocytes was documented following linsitinib treatment on both days 5 and 9 (Figure 6M). Considering linsitinib’s known affinity for the insulin receptor (31), which also plays a role in adipogenesis, we conducted additional experiments in insulin-free media to show the insulin-independent effects of IGF-1R inhibition on adipogenesis. Despite the overall reduction in adipogenesis due to the absence of insulin, a critical factor in adipocyte differentiation, linsitinib treatment still resulted in a dose-dependent inhibition of adipogenesis (Figure 6N). To confirm that the decrease in adipogenesis associated with linsitinib treatment is due to inhibition of IGF-1R signaling, we performed Western blots to demonstrate that treatment with linsitinib is associated with a dose-dependent decrease in IGF-1R phosphorylation on days 5 and 9 (Figure 6O). These findings emphasize the critical role of IGF-1R signaling in the adipogenic process of TED orbital fibroblasts, highlighting how linsitinib’s influence on this pathway markedly affects adipogenesis, independent of insulin signaling.

## Discussion

In this study, we utilized snRNA-Seq to explore the cellular landscape of orbital fat in TED, providing the first in vivo evidence to our knowledge of increased adipogenesis linked to this condition. We observed a significant rise in the number of adipocytes within the orbital fat of patients with TED, alongside differential expression of components in the IGF-1 signaling pathway. Specifically, orbital fibroblasts in patients with TED were enriched with IGF-1 pathway components, contrasting with the decreased pathway activity observed in TED adipocytes. These observations are consistent with previous findings of increased surface display of IGF-1R on orbital fibroblasts of patients with TED compared with control patients (19, 32) and extend our previous in vitro work (18), reinforcing the hypothesis that disrupted IGF-1 signaling is crucial in TED pathogenesis.

IGF-1R has been shown to function as an autoantigen in TED (32, 33) and to coimmunoprecipitate with TSHR in orbital fibroblasts. Inhibition of IGF-1R activation decreases signaling downstream of TSHR activation (19, 32). IGF-1 produced by orbital fibroblasts may have varying roles depending on the target cells. In orbital fibroblasts, IGF-1 increases hyaluronan and chondroitin sulfate (19, 32, 33). Orbital fibroblasts do not exist in isolation in the orbit, but rather orbital fat is enriched in immune cell populations, including monocytes/macrophages, NK cells, and B and T lymphocytes. IGF-1 produced by orbital fibroblasts is likely also signaled to these cell types in a paracrine manner and has been shown to promote lymphocyte proliferation and the production of proinflammatory cytokines by immune cells (33).

The role of IGF-1 signaling in orbital adipocytes has not been fully explored; however, IGF-1 signaling has been shown to be essential in adipogenesis of 3T3-L1 cells (34). In the mouse epigonadal fat pad, IGF-1 stimulation appears to decrease *Igf1* expression, which may help to explain the finding of decreased IGF gene expression as orbital fibroblasts transition to adipocytes (35). We used linsitinib, a small-molecule IGF-1 receptor inhibitor that is in clinical trials for TED to demonstrate that pharmacologic inhibition of IGF-1R function in orbital fibroblasts decreases adipogenesis in an insulin-independent manner. Although IGF/IGF1R signaling declines in adipocytes compared with fibroblasts in TED, linsitinib may be effective in controlling early stages of adipogenesis, particularly in targeting precursor fibroblasts before differentiation to adipocytes. Thus, potential mechanisms of IGF-1R inhibition to treat TED may include decreasing adipogenesis as well as changing local cytokine expression patterns in immune cells.

Highlighting the complexity of IGF signaling dysfunction in TED, we found an upregulation of *IGFBP5* and downregulation of *IGFBP6* in TED orbital fibroblasts, which diminished as the fibroblasts differentiated into adipocytes (Figure 5B). This is consistent with previous work indicating that IGFBP5 is upregulated alongside IGF signaling in vascular smooth muscle cells (36).

Additionally, we found that TED immune cells exhibit higher levels of *IGFBP6* expression compared with those from controls, indicating that IGF1 pathway dysfunction in the orbit is not limited to fibroblasts and adipocytes (Supplemental Table 3). The role of IGFBP5 in modulating IGF signaling is complex, potentially inhibiting (37, 38) or enhancing (39, 40) IGF signaling, depending on calcium concentrations in the surrounding environment (41). Both IGFBP5 and IGFBP6 bind IGF2 more strongly than IGF1 and possess IGF-independent functions, including effects on apoptosis, cell adhesion, and angiogenesis (42–44). Further studies are needed to better describe the levels of IGF1 and IGFBPs in orbital fat to delineate how IGFBP expression modulates IGF1 signaling in TED. Moreover, investigating the potential IGF-independent roles of IGFBPs in orbital fibroblasts will be crucial for a more comprehensive understanding of their effect on the disease’s pathophysiology.

Our analyses also highlight two potential mechanisms driving adipocyte accumulation in TED: enhanced differentiation of orbital fibroblasts into adipocytes and increased proliferation of preexisting adipocytes. Notably, TED adipocytes exhibited a downregulation in the expression of several IGF pathway genes, such as *IGFBP5*, *IGFBP6*, *IGFL4*, *IGFBP2*, and *IGF1R*, relative to fibroblasts. This reduced IGF signaling is likely a key factor contributing to TED progression and indicates a shift in cellular signaling pathways that warrants further exploration.

In TED orbital fibroblasts, we observed not only the upregulation of adipocytes-enriched genes but also higher expression levels of genes that regulate adipogenesis, including *CEBPB* and *CEBPD* (23), *FABP4* (24), *RASD1* (29), and *APOE* (28). However, we detected no changes in other adipogenic genes, such as *FABP5* (25), *PLIN2* (26), and *APOC1* (27). The specific roles of these genes in adipogenesis require further investigation. Additionally, we identified other genes in TED fibroblasts that may facilitate adipogenesis (Supplemental Table 8), such as *NFATC2,* which can regulate phospholipase A2 (45), a potential adipogenesis target (46), and induces adipocytes in 3T3 cells (47, 48). ZBTB16 also induces adipogenesis in vitro (49) and is recognized as a potential adipogenic factor (50). Furthermore, silencing *LRP1B* in rodents has been demonstrated to inhibit adipogenesis (51).

The use of snRNA-Seq in our study offers a refined approach for profiling the cellular composition of orbital fat in TED. This choice is particularly advantageous over single-cell RNA-Seq (scRNA-Seq) due to its enhanced capacity for capturing adipocyte populations, which are typically challenging due to their physical properties (52–54). Interestingly, our results do not fully align with scRNA-Seq data previously published by Li and colleagues (55), where adipocytes were not identified, a discrepancy likely due to selective loss of these cells. Furthermore, the samples profiled by Li and colleagues were isolated from patients with generally higher clinical activity scores (CASs) and greater severity, requiring decompression for compressive optic neuropathy, which may account for the differences in the distribution of immune cells described (55).

In this snRNA-Seq analysis, we did not detect SMA^+^ myofibroblasts, which are considered critical for fibrosis in TED (56–60). These cells have been described in vitro following the treatment of orbital fibroblasts with TGF-β (60, 61). The absence in our samples could be attributed to the relatively low CASs of the sampled patients, suggesting a correlation between lower CASs and less active fibrosis due to the absence of this cell population. Alternatively, the lack of SMA^+^ myofibroblasts might be a sampling artifact, as these cells may not reside in the orbital fat but rather in other orbital tissues like extraocular muscles, Tenon’s fascia, or muscle sheaths, which are not routinely biopsied in TED. Profiling additional orbital fat samples from patients at various disease stages and activity levels may provide further insights into the fibroblast populations mediating fibrosis in TED. This absence might also indicate that in vivo conversion of preadipocyte fibroblasts into SMA^+^ myofibroblasts does not occur in patients with a CAS of less than or equal to 3.

While tissue culture models have advanced our understanding of TED by allowing for manipulations of cell signaling pathways to uncover disease mechanisms, the use of serially passaged cells has inherent limitations. Our study’s snRNA-Seq data provide additional validation for the role of adipogenesis in TED and suggest the therapeutic potential of IGF-1R inhibition. However, the cross-sectional design of our study limits our ability to collect longitudinal data, which could offer deeper insights into the disease’s progression. Additionally, our dataset involves a limited number of patients with relatively low CASs, all presenting for surgery during the study period. Future studies should aim to include a broader patient cohort, encompassing various stages of TED, to more comprehensively elucidate the progression and cellular dynamics within the orbit.

In particular, further studies of the role of immune cells in the progression of TED; the potential interplay among inflammation, adipogenesis, and adipocyte proliferation on TED pathology; and the impact of thyroid hormone regulation of IGF-1 signaling versus treatment effects should be undertaken.

A potential caveat of this study is the variation in age between patients with TED and control patients, which may influence the observed adipogenesis and orbital fat expansion. Additionally, the limited smoking history data, particularly among patients with TED, could affect the generalizability of our findings, given the established role of smoking in TED pathogenesis (62). In addition, immune cell types were identified based on gene expression without surface marker validation (63, 64), which can make conclusive identification of some subtypes challenging. Additionally, the expression profiles observed may be specific to tissue-resident immune cells, potentially limiting the generalizability of comparisons to reference immune populations.

## Methods

### Sex as a biological variable.

Our study examined male and female human samples (Supplemental Table 1), and we did not observe any sex-dimorphic effects in this study. Demographic details are shown in Supplemental Table 1.

### Cell culture.

Orbital fibroblast cell lines were derived from the retrobulbar fat of patients with TED and control patients as previously described (65, 66). Briefly, tissue explants were obtained from patients undergoing surgical decompression for TED or excision of prolapsed orbital fat, which has been shown to be retrobulbar fat, in control patients (65). Patients with TED were inactive, with a CAS of less than 4, at the time of surgery (67). The explants were placed on the bottom of culture plates and covered with Eagle’s medium containing 10% FBS, antibiotics, and glutamine, and then incubated at 37°C, 5% CO_2_, in a humidified environment. The culture media was refreshed weekly until cell confluence was achieved. The resulting fibroblast monolayers were passaged serially by treatment with TrypLE (12604039, Thermo Fisher Scientific), and maintained in liquid nitrogen storage for future use.

For adipogenesis induction, orbital fibroblasts were induced to undergo adipogenesis as previously described (66). Briefly, fibroblasts between passages 4 and 8 were seeded on plastic tissue culture plates until near confluence in DMEM containing 10% FBS and antibiotics. Adipogenic differentiation was initiated by treating the cells with adipogenic medium composed of DMEM:F-10 (1:1, 11320033, Thermo Fisher Scientific) supplemented with 3% FBS, 100 nmol/L insulin (12585014, Thermo Fisher Scientific), 1 μmol/L dexamethasone (D4902, MilliporeSigma), and, for the first week only, 1 μmol/L rosiglitazone (R2408, MilliporeSigma) and 0.2 mmol/L IBMX (13630S, Cell Signaling Technology). Linsitinib (T69071, TargetMol) was added to the adipogenic media at the indicated concentration during the first week only. The medium was replaced every other day for the first week and the culture was continued in this medium for a total of 9 days. Control cultures were treated with DMEM:F-10 (1:1), supplemented with 3% FBS. Differentiation was observed using a Nikon microscope, with each time point replicated in triplicate.

### Quantification of adipocytes.

On days 0, 5, and 9 of the experiment, cells were washed with PBS followed by fixation with 4% paraformaldehyde in PBS for 10 minutes at room temperature. Cells were then washed with PBS or stored in PBS at 4°C. For adipocyte staining, cells were first washed with 60% isopropanol. Staining was then performed with freshly prepared 0.3% (w/v) Oil red O (MilliporeSigma) at room temperature for 15 minutes. After the staining process, cells were rinsed with 60% isopropanol and counterstained with hematoxylin (MilliporeSigma) for 5 minutes, followed by a wash with PBS. Cells were imaged by a masked study team member using a Keyence BZ-X710 microscope. For each treatment replicate, 5 high-power images were captured. Cell counts and lipid vacuole measurements were performed by a masked study team member using ImageJ (NIH) (68). Adipocytes were identified as Oil Red O^+^ cells, and nuclei in cells that did not stain with Oil Red O, indicative of fibroblasts or preadipocytes, were excluded from the adipocyte count. Data are presented as mean with SD.

### Western blot.

Cellular protein was collected in RIPA buffer (R0278, MilliporeSigma) containing ReadyShield Protease and Phosphatase Inhibitor Cocktail (PPC- 2020, MilliporeSigma) on days 0, 5 and 9 of the experiment. The protein extracts were subjected to quantification using the DC Protein Assay Reagents (5000116, Bio-Rad). The RIPA protein samples were mixed with 4X protein sample buffer (1610747, Bio-Rad) containing 5% 2-mercaptoethanol (1610710, Bio-Rad) and heated at 95°C for 5 minutes to denature. Proteins were loaded onto 10% bis-Tris gels and transferred to Immun-Blot PVDF membranes (1620177, Bio-Rad), which were then blocked in 5% blocking grade skim milk (170-6404, Bio-Rad). Antibodies specific for phospho–IGF-1R (3918, Cell Signal Technology), IGF-1R (3027, Cell Signal Technology), and actin (5125, Cell Signal Technology) were used at a dilution of 1:1,000. Antibody recognizing Annexin A1 (ANXA1; 71-3400, Thermo Fisher), γ cystathionase (CTH; 60234-1, ProreintTech) and PDZ domain containing ring finger 4 (PDZRN4; H00029951-B01P, Novus Biologicals) were used at a dilution of 1:2,500. The membranes were incubated with the appropriate primary antibody overnight followed by horseradish peroxidase–conjugated anti-rabbit (7074, Cell Signaling Technology) secondary antibody at a dilution of 1:2,500 for an hour at room temperature. The signal was detected with a chemiluminescence detection system (Thermo Scientific). Blots were imaged with an iBright CL1500 Imaging System (Thermo Fisher Scientific), and band intensity was reported as arbitrary densitometric units using ImageJ software (NIH).

### snRNA-Seq.

Human retrobulbar orbital fat was obtained from patients with TED undergoing orbital decompression or control patients undergoing routine resection of prolapsed orbital fat (Supplemental Table 1), which is intraconal fat (65), using a protocol approved by the Johns Hopkins University Institutional Review Board and following the tenets of the Declaration of Helsinki. Patients with TED were inactive at the time of the decision to proceed with surgery, based on a CAS of less than 4 (Supplemental Table 1). Nuclei from retrobulbar orbital fat were isolated using the methods described in the 10x Genomics Sample Preparation Demonstrated Protocol (10x Genomics). Briefly, cells were washed with chilled PBS and lysed in chilled lysis buffer consisting of 10 mM Tris-HCl, 10 mM NaCl, 3 mM MgCl_2_, and 0.1% Nonidet P40 Substitute (MP Biomedicals) in nuclease-free water at 4°C. Cells were scraped from the plate bottom and centrifuged at 500 RCF for 5 minutes at 4°C. Cells were washed twice in nuclei wash and resuspension buffer consisting of PBS with 0.1% BSA and 0.2 U/μL RNase inhibitor (N2615, Promega). Cells were passed through a 50 μm CellTrics filter (NC9491906, Sysmex) and centrifuged at 500 RCF for 5 minutes at 4°C before resuspension in wash and resuspension buffer. Isolated nuclei were counted manually via hemocytometer with Trypan Blue staining, and nucleus concentration was adjusted following the 10x Genomics guidelines. A total of 10 libraries was generated using the 10x Genomics Chromium Single Cell system, V3.1 chemistry, per manufacturer’s instructions. A couple of biological groups were run with technical replicates (Supplemental Table 1). Libraries were sequenced on Illumina NovaSeq 6000 with 500 million reads per sample. Sequencing data were preprocessed through the Cell Ranger pipeline (10x Genomics, Cellranger v5.0.0) with default parameters, using GRCh38-2020-A genome with *include-introns*.

Matrix files generated from the Cellranger run were used for subsequent analysis as described previously (18, 69). Briefly, Seurat V3 (70) was used to perform downstream analysis, only including cells with more than 500 genes, and 1,000 unique molecular identifiers (UMI) to process control and treated samples separately. Seurat SCTransform function was used to normalize the dataset (71), and UMAP was used to reduce dimensions derived from the Harmony output (72). Individual cell types were first identified by cross-referencing to the ASCOT database (73). To identify immune cell types, gene expression profiles from each cluster were compared with publicly available databases (73, 74), and marker genes were used to assign likely identities to each cluster.

Differential gene tests on the scRNA-Seq datasets were initially performed using Seurat v3.15 *FindAllMarkers* function (*test.use* = *“wilcox”, logfc.threshold* = *0.5, min.pct* = *0.2)*. Top differential genes (adjusted *P* value < 0.05, fold change > 0.5) in each microglial cluster were used as input for GO pathway analysis using ClusterProfiler (v3.12.0) (75). Pseudotime analysis was performed using Monocle v3 (76), and significant genes (*q* < 0.001) along pseudotime trajectory were used to run KEGG analysis as described above. Genes that were involved across KEGG signaling pathways were then used to identify signaling pathways that are specific to adipocyte differentiation.

### Statistics.

Statistical analyses were conducted using GraphPad Prism. Data are presented as the mean ± SD. To determine statistical significance, an unpaired 2-tailed *t* test was used for comparisons between 2 groups. For experiments involving more than 2 groups, a 1-way or 2-way ANOVA was performed, as appropriate, followed by Tukey’s or Šidák’s multiple comparison test. A *P* value of less than 0.05 was considered statistically significant. Specific details of each statistical test are provided in the relevant figure legends.

### Study approval.

This study was carried out following a protocol approved by the Johns Hopkins University Institutional Review Board and following the tenets of the Declaration of Helsinki. Informed consent was obtained prior to the collection of patient tissue for all experiments.

### Data availability.

Values underlying graphed date can be viewed in the supporting data values file. All other data reported in this paper will be shared by the authors upon request due to IRB policy against open-access sharing of human subjects data. No custom code was used in this study.

## Author contributions

FR designed research studies. DWK, SK, JH, and KB conducted experiments. DWK, SK, and FR analyzed data. EL, NM, SB, and FR provided reagents. DWK, SB, and FR acquired funding. DWK and FR wrote the manuscript. DWK, SB, and FR provided critical review of the manuscript.

## Supplementary Material

Supplemental data

Unedited blot and gel images

Supplemental tables 1-8

Supporting data values

## Version 1 12/20/2024

Electronic publication
