# Supplementary figures and images for: Transcriptomic profiling of thyroid eye disease orbital fat demonstrates differences in adipogenicity and IGF-1R pathway
Source: JCI Insight. 2024 Dec 20;9(24):e182352. doi: 10.1172/jci.insight.182352 (PMC11665563; doi:10.1172/jci.insight.182352)

A

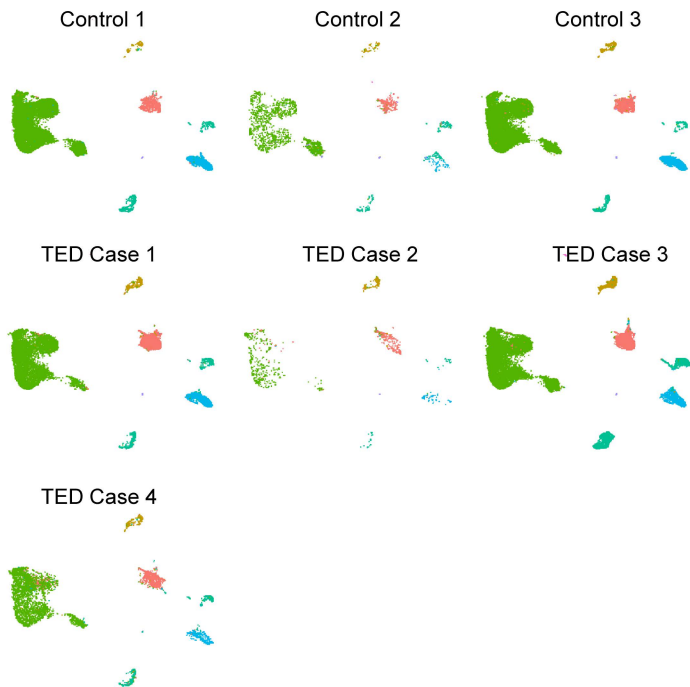

B

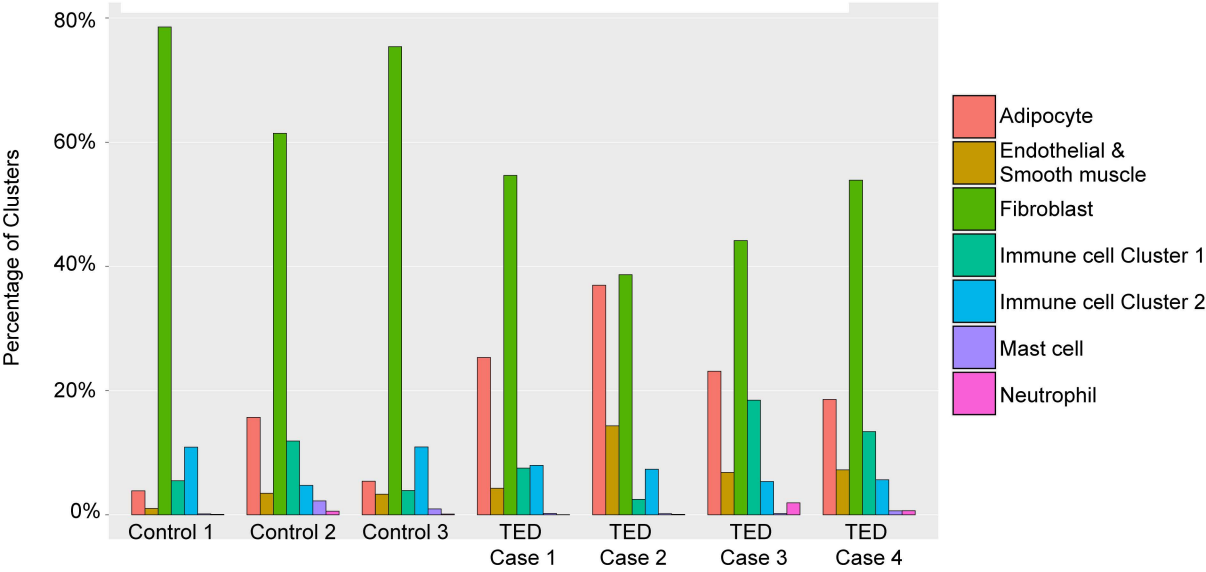

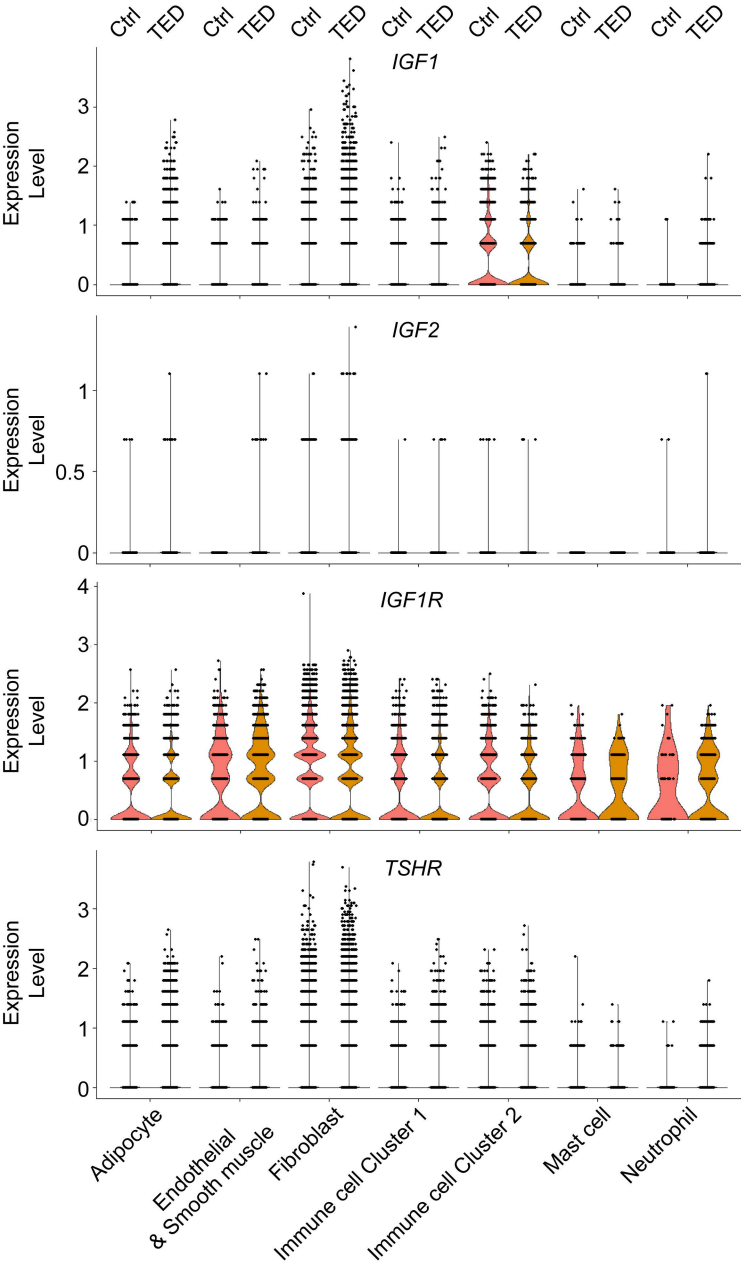

Expression  
Low High

*PDE3B*

*PLIN1*

Ctrl

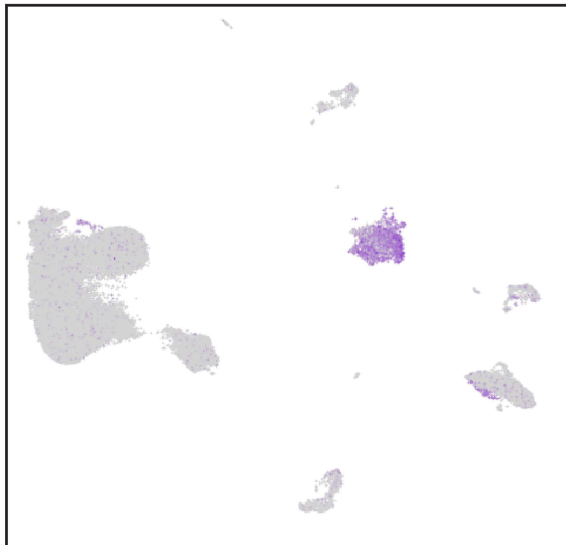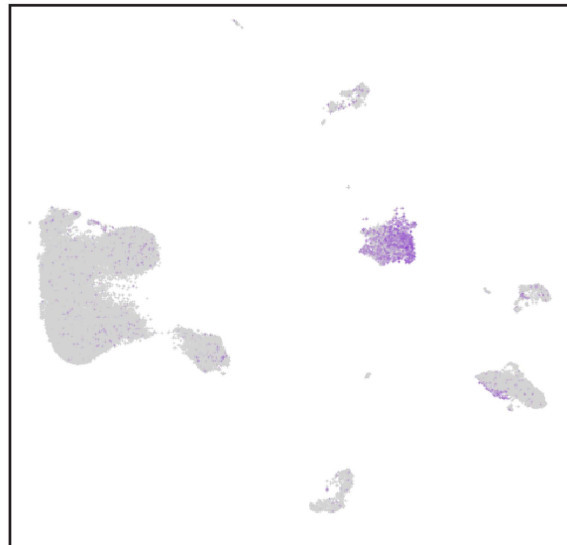

TED

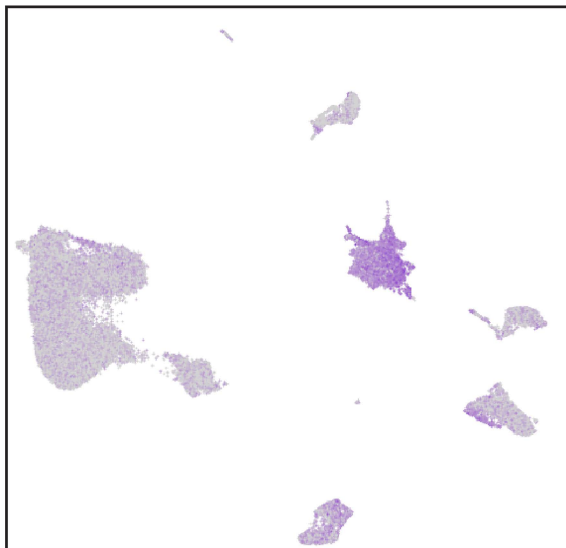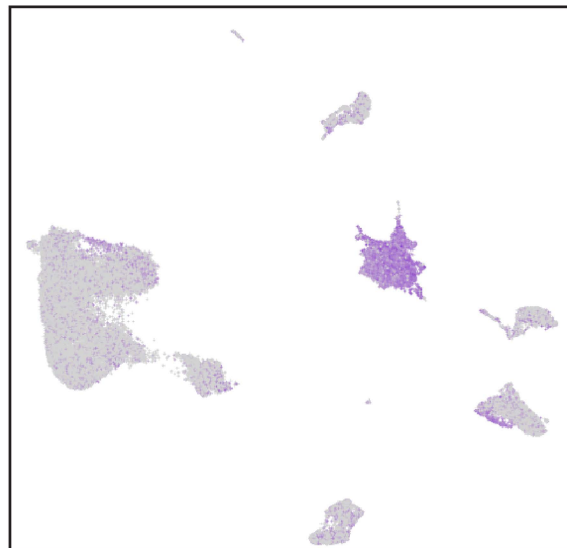

## Immune Cells

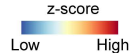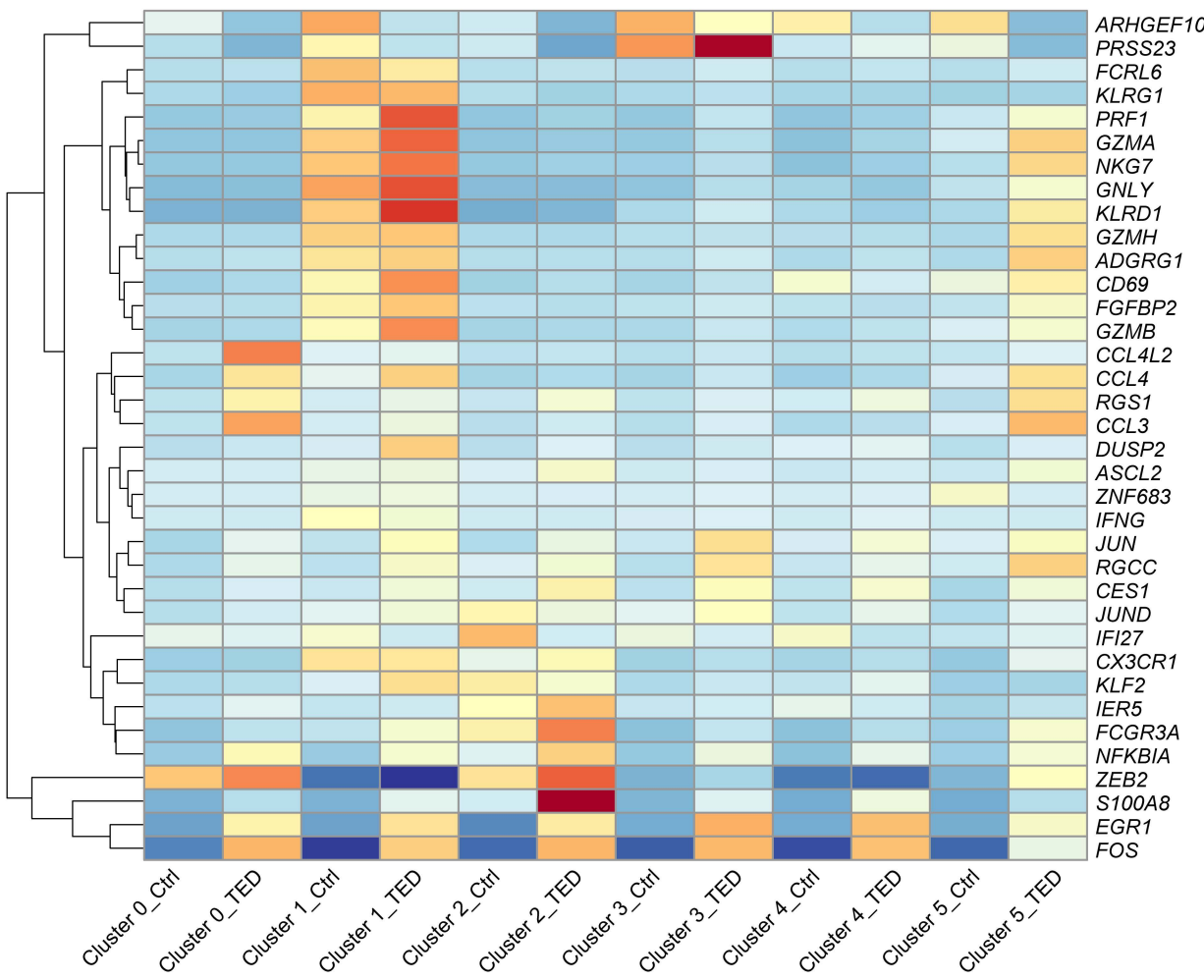

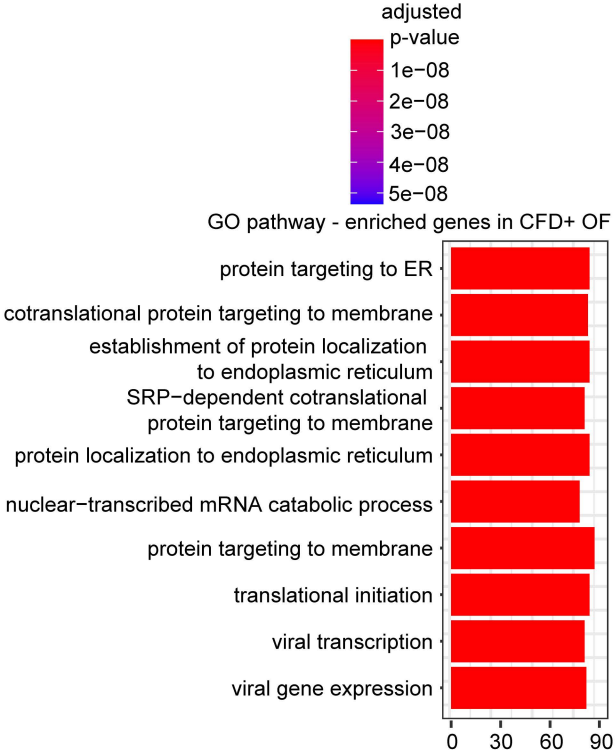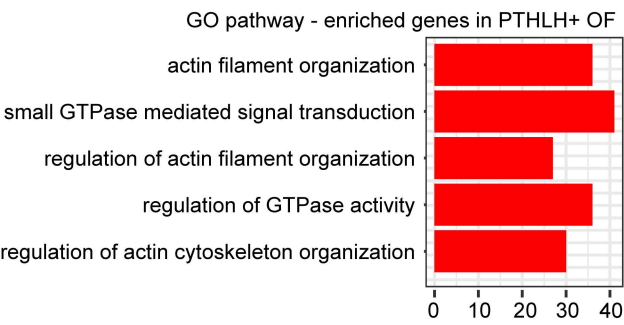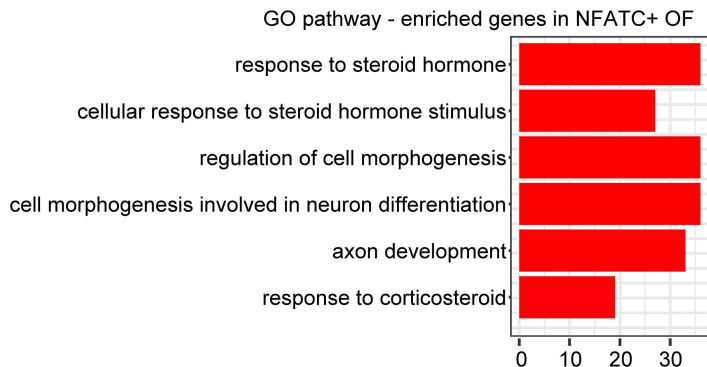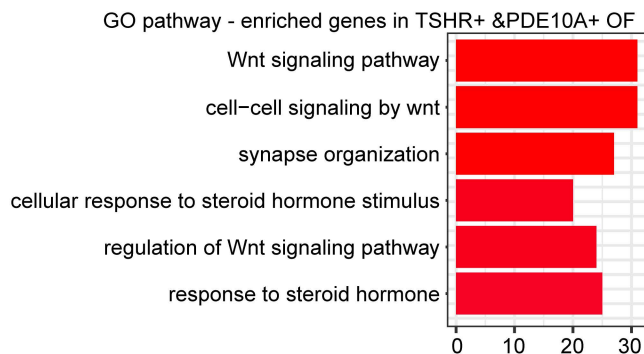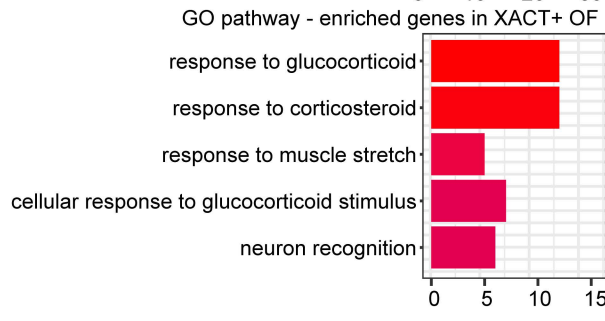

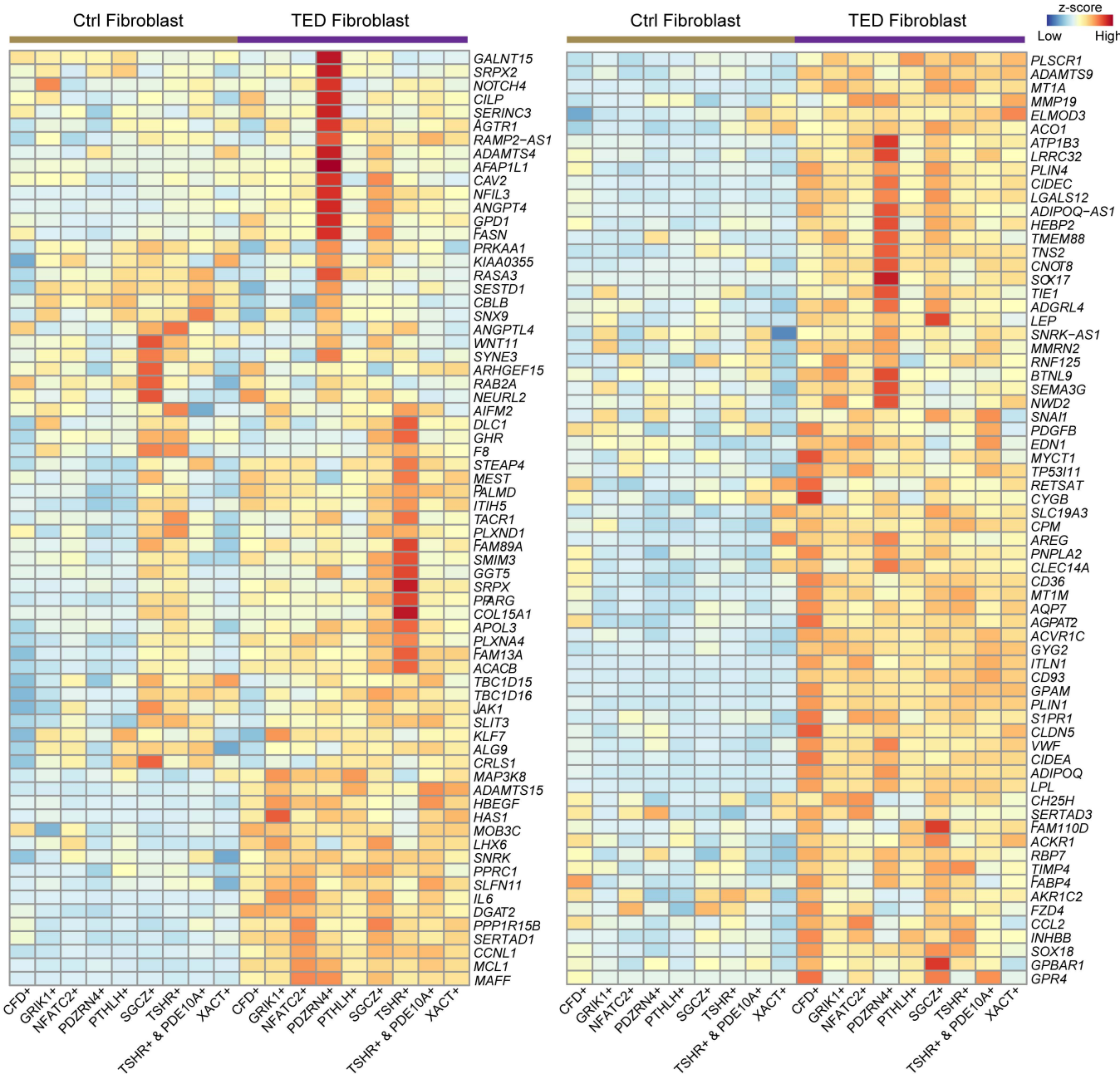

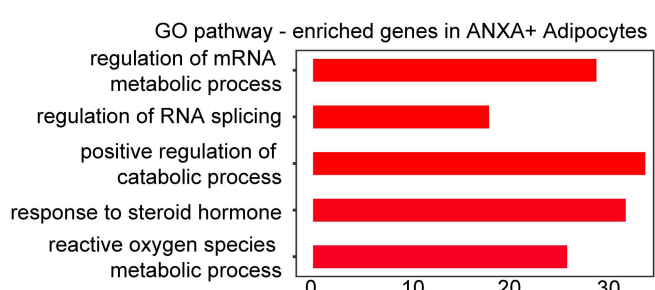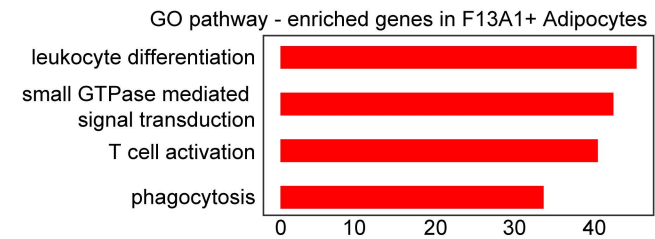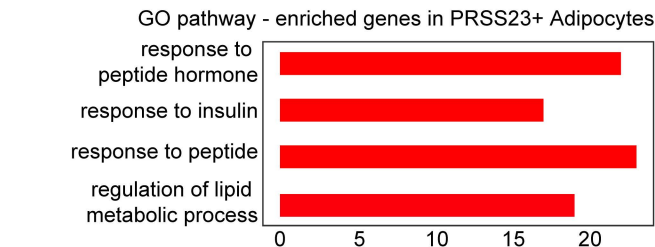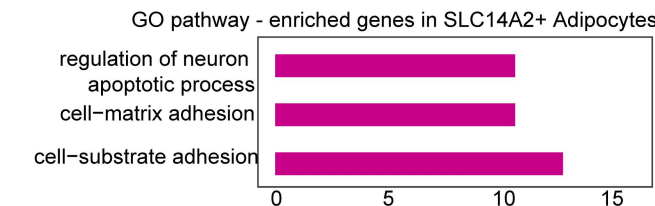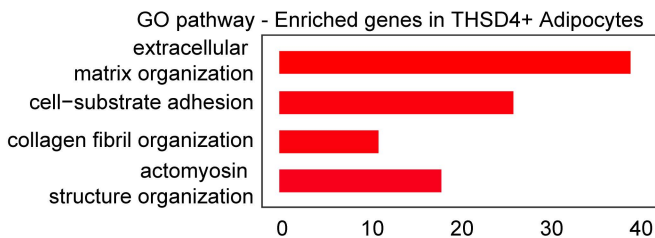

adjusted p-value

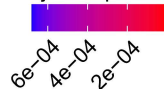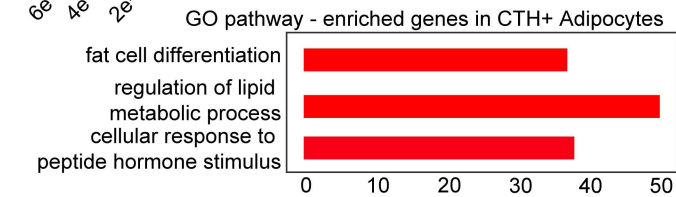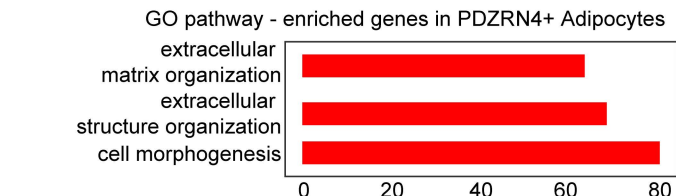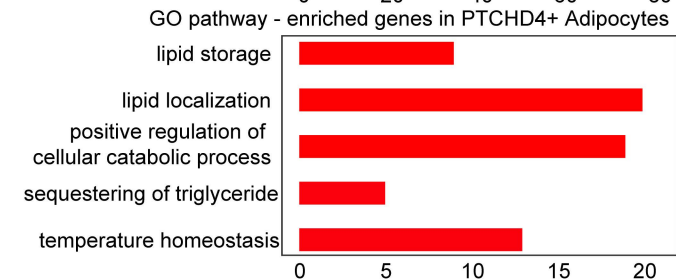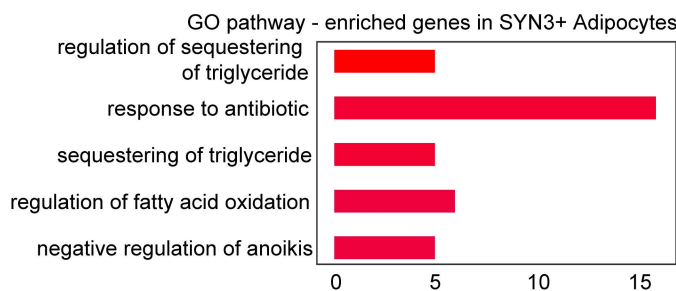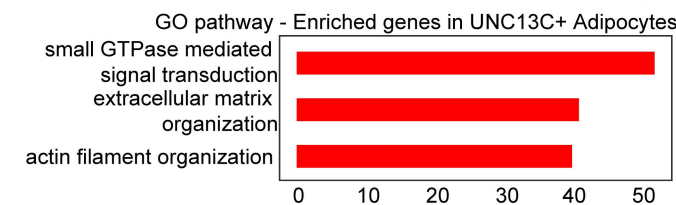

Supplement: Supplemental data [file jciinsight-9-182352-s092.pdf]
