# Supplementary material for: Transcriptomic profiling of thyroid eye disease orbital fat demonstrates differences in adipogenicity and IGF-1R pathway
Source: JCI Insight. 2024 Dec 20;9(24):e182352. doi: 10.1172/jci.insight.182352 (PMC11665563; doi:10.1172/jci.insight.182352)
Supplement: Unedited blot and gel images [file jciinsight-9-182352-s093.pdf]

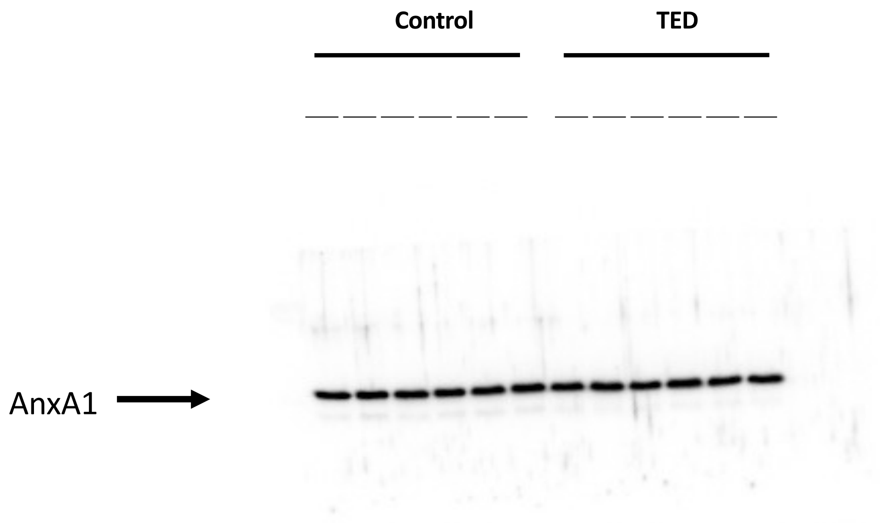
Control
TED
AnxA1

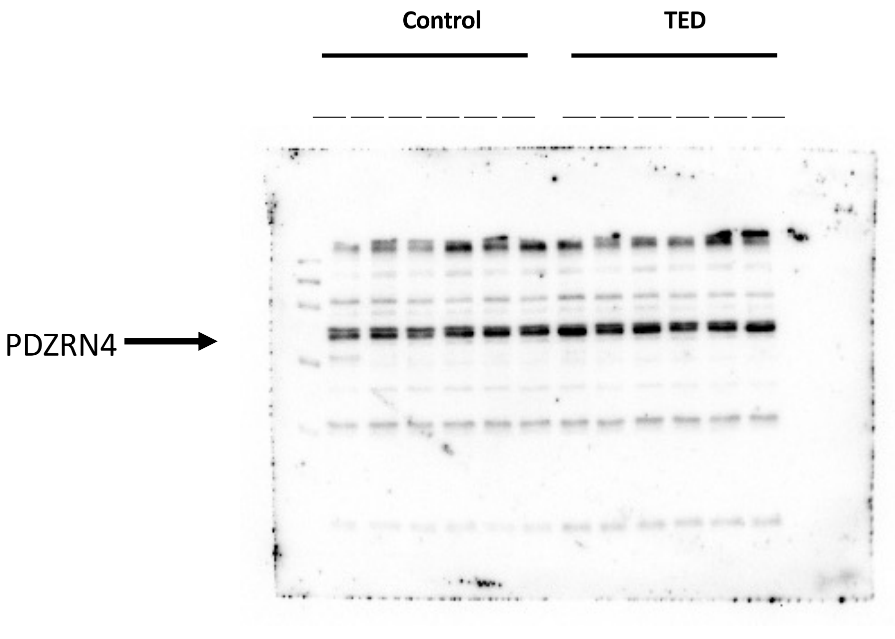
Control
TED
PDZRN4

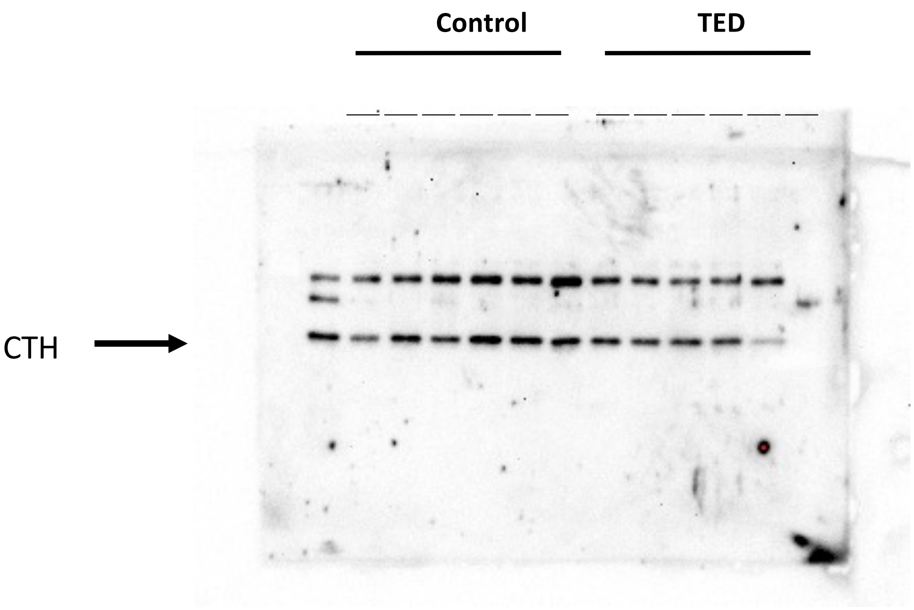
Control
TED
CTH

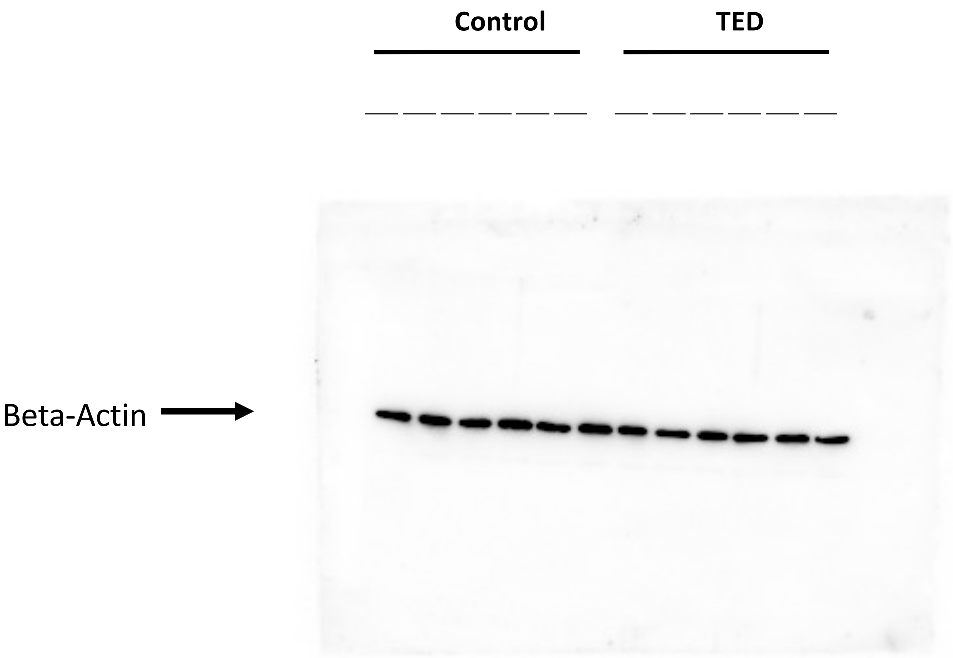
Control
TED
Beta-Actin

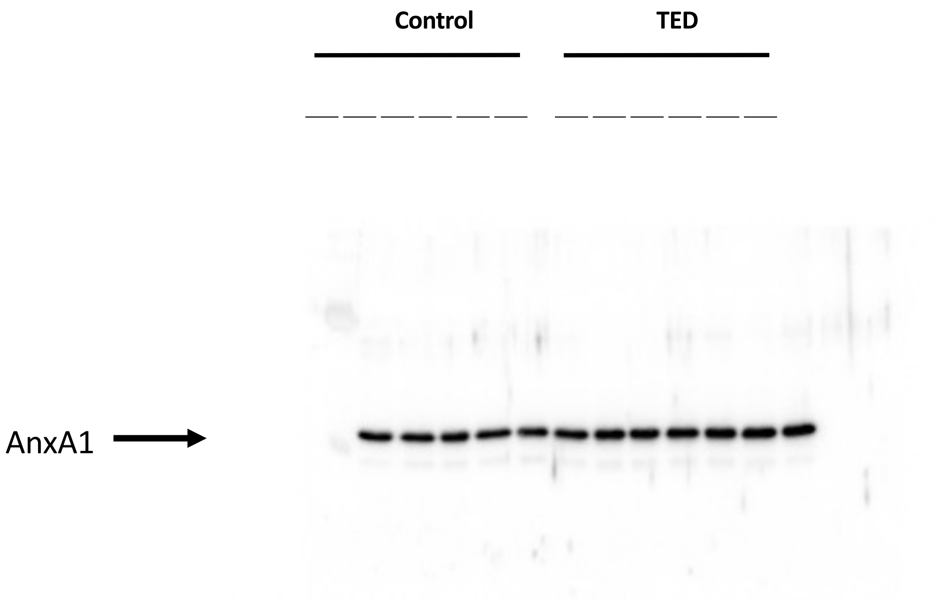
Control
TED
AnxA1

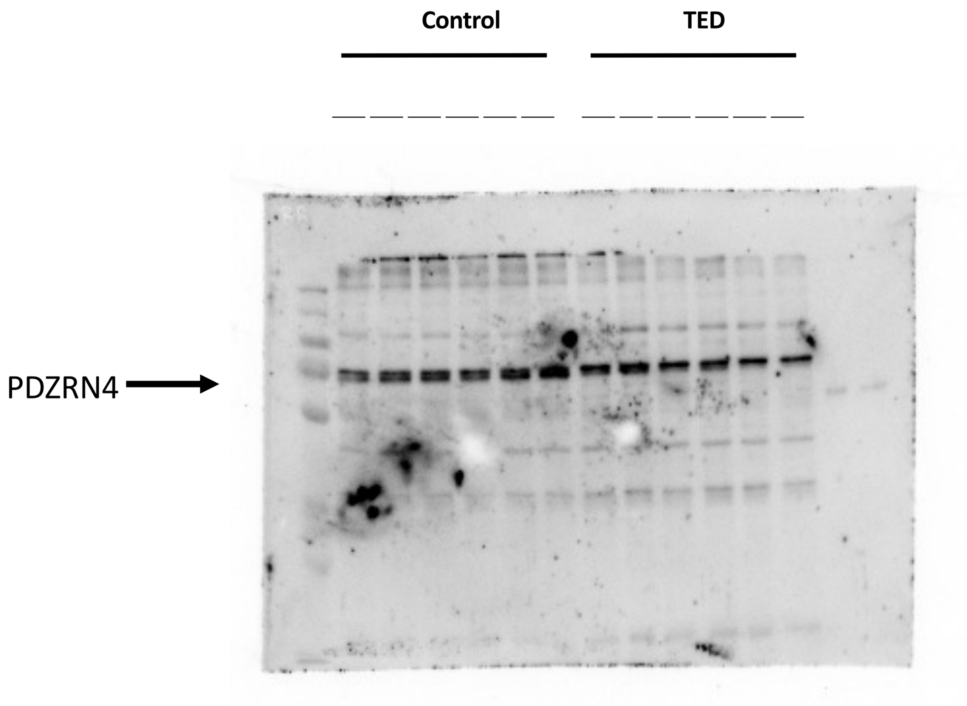
Control
TED
PDZRN4

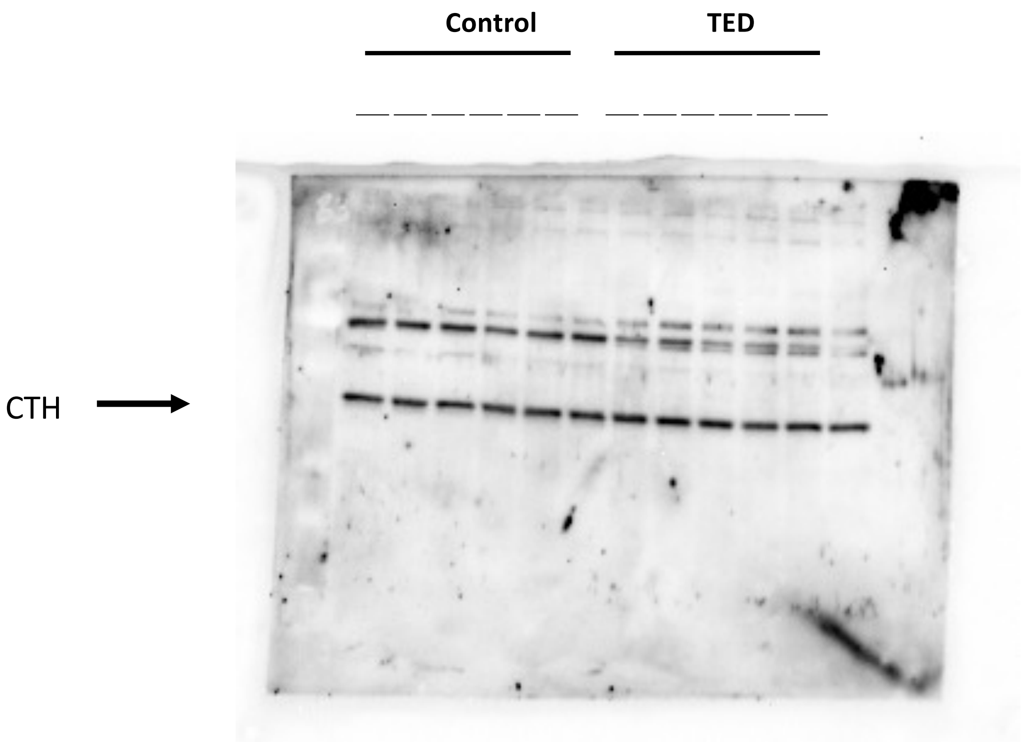
Control
TED
CTH

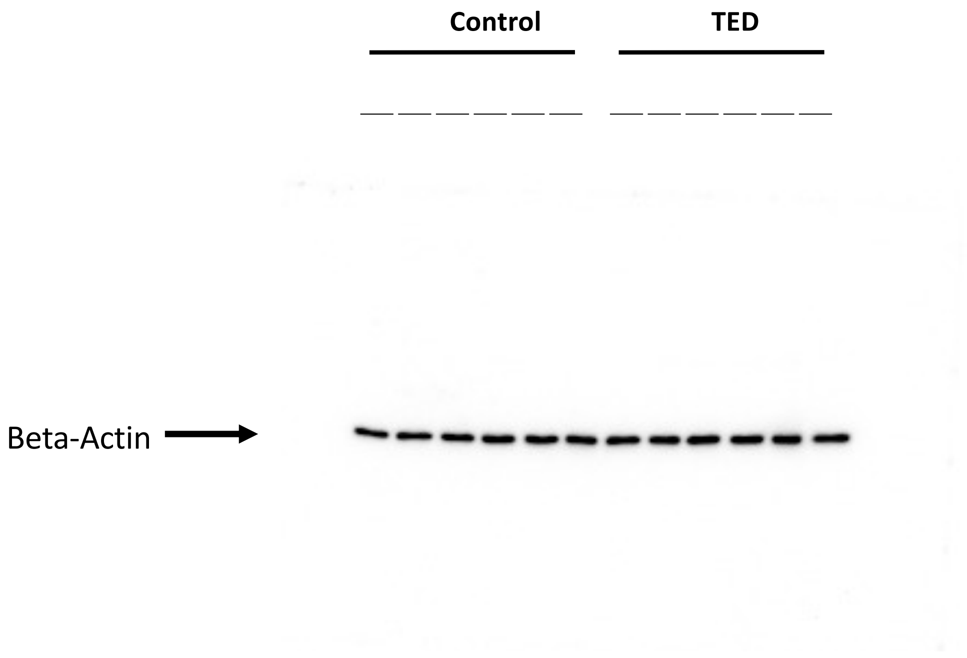
Control
TED
Beta-Actin

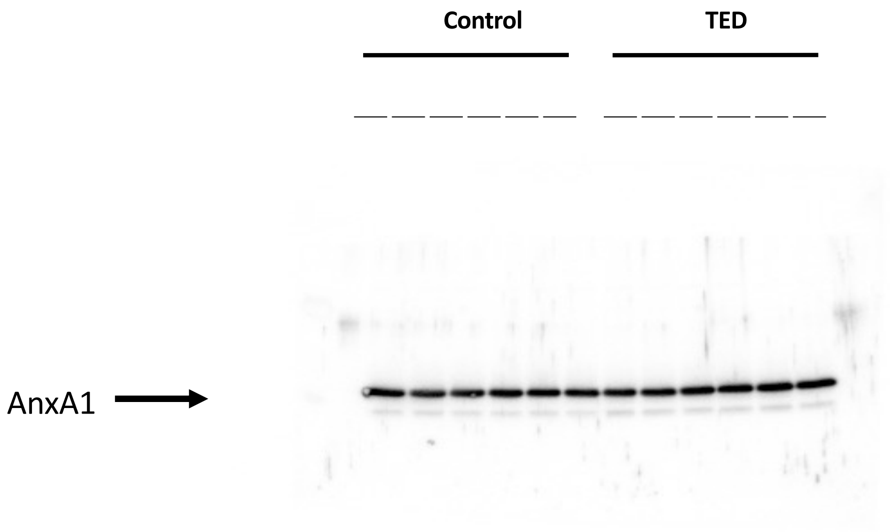
Control
TED
AnxA1

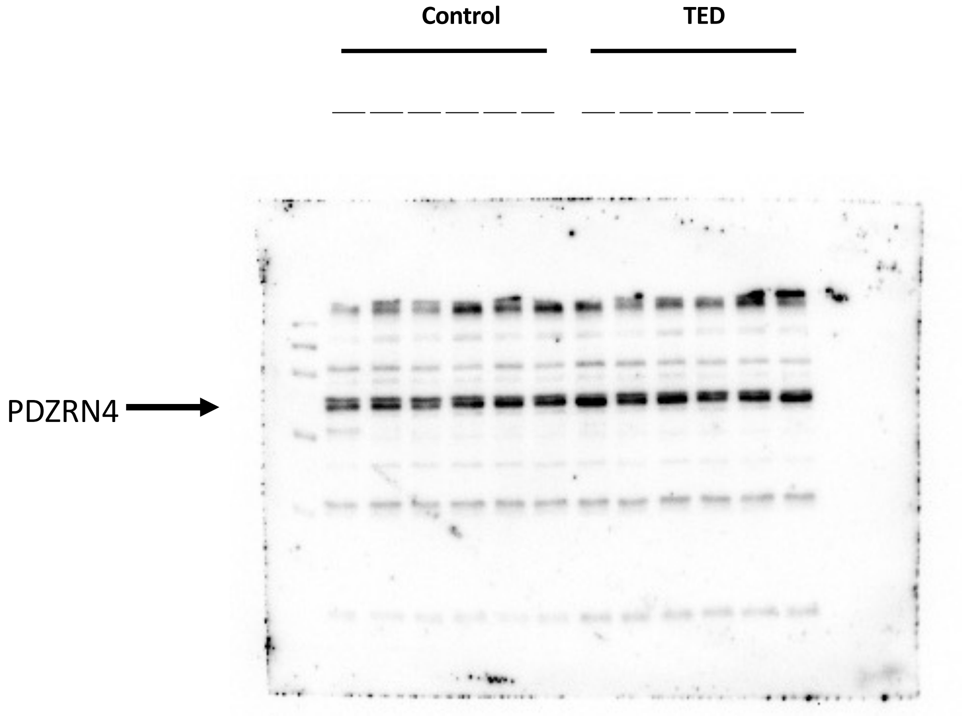
Control
TED
PDZRN4

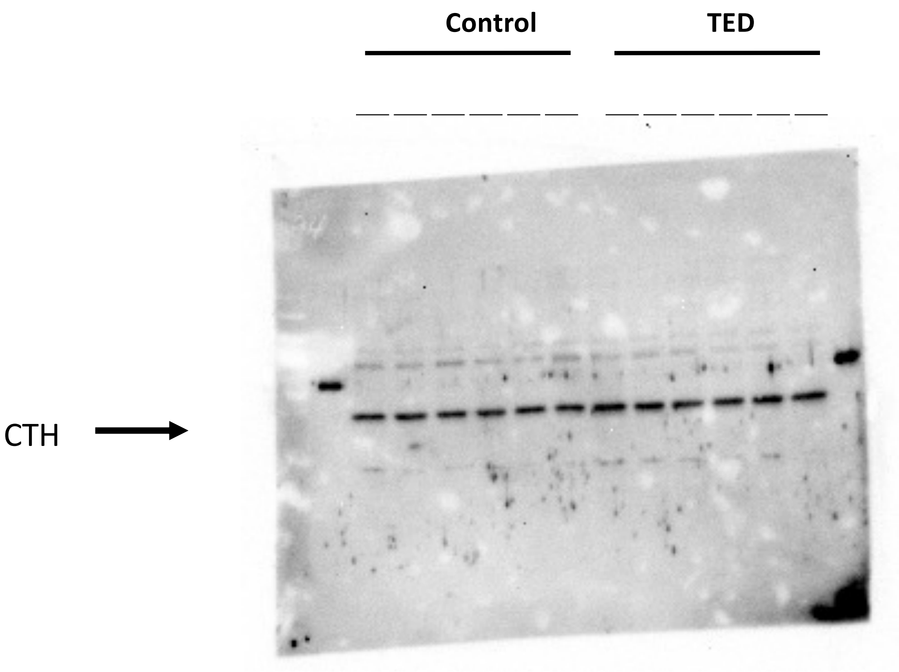
Control
TED
CTH

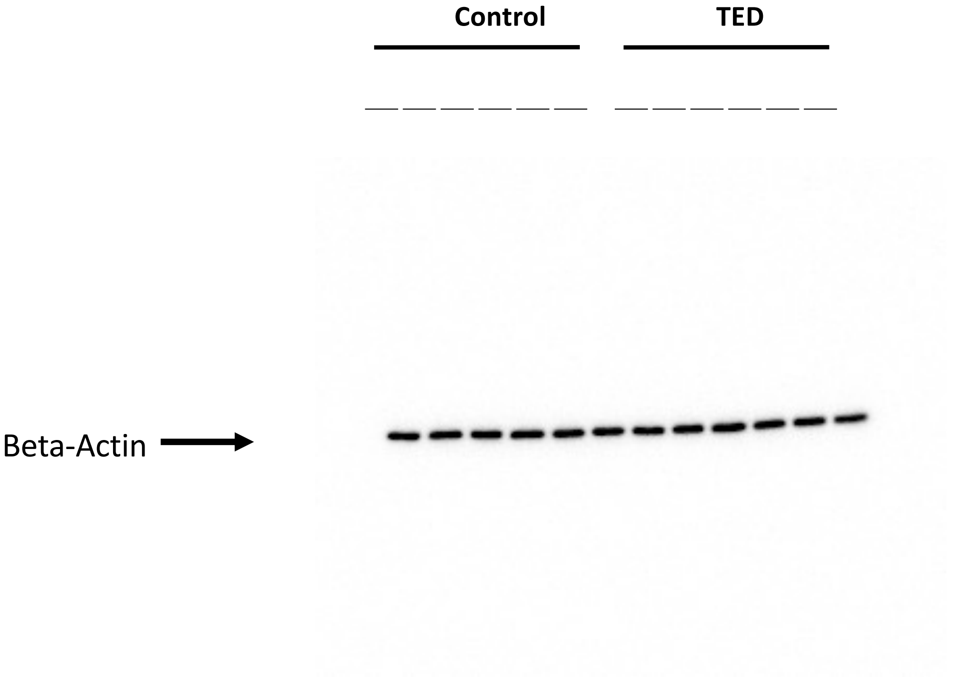
Control
TED
Beta-Actin

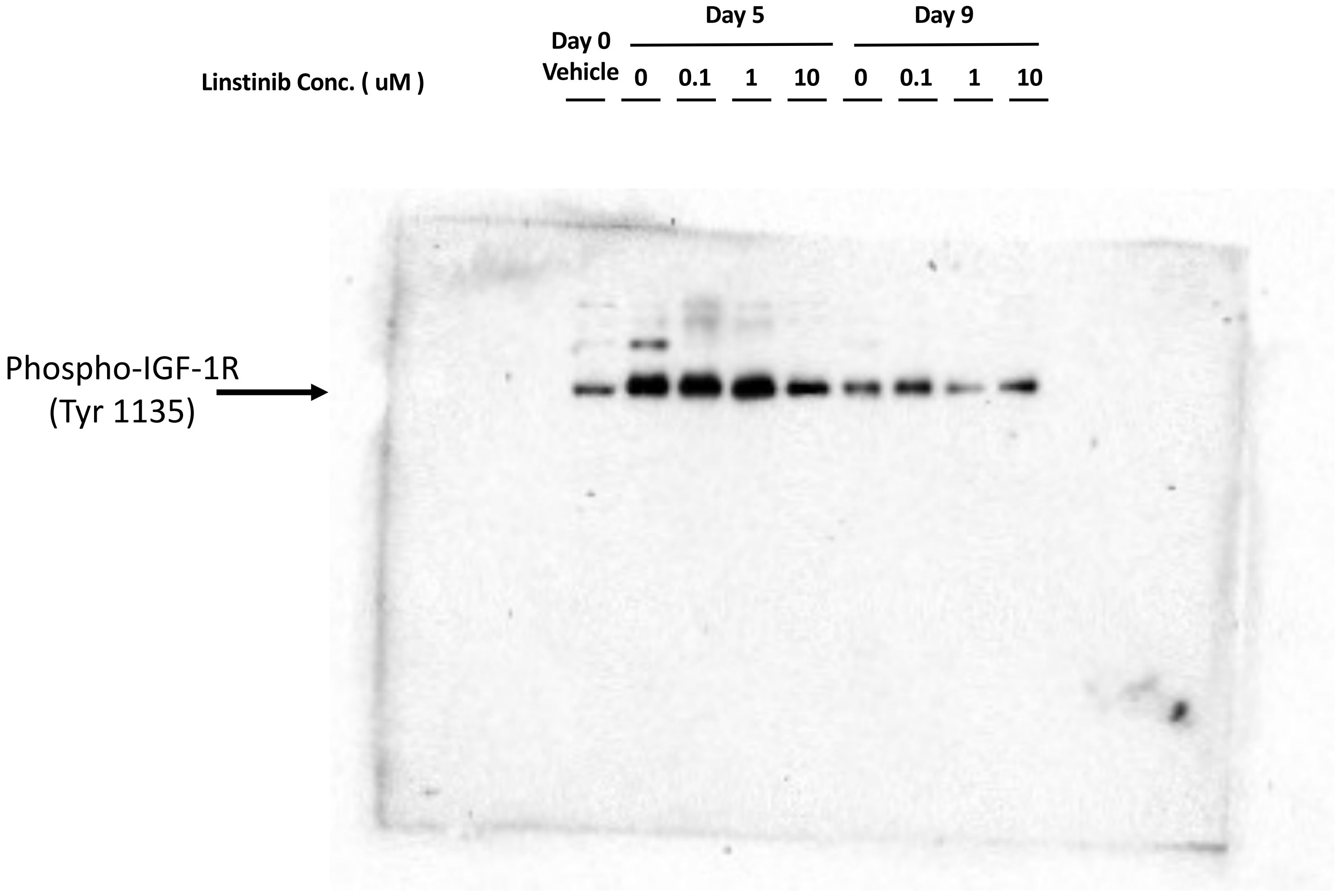
Day 5
Day 9
Day 0
Vehicle
Linstinib Conc. (uM)
0
0.1
1
10
0
0.1
1
10
Phospho-IGF-1R
(Tyr 1135)

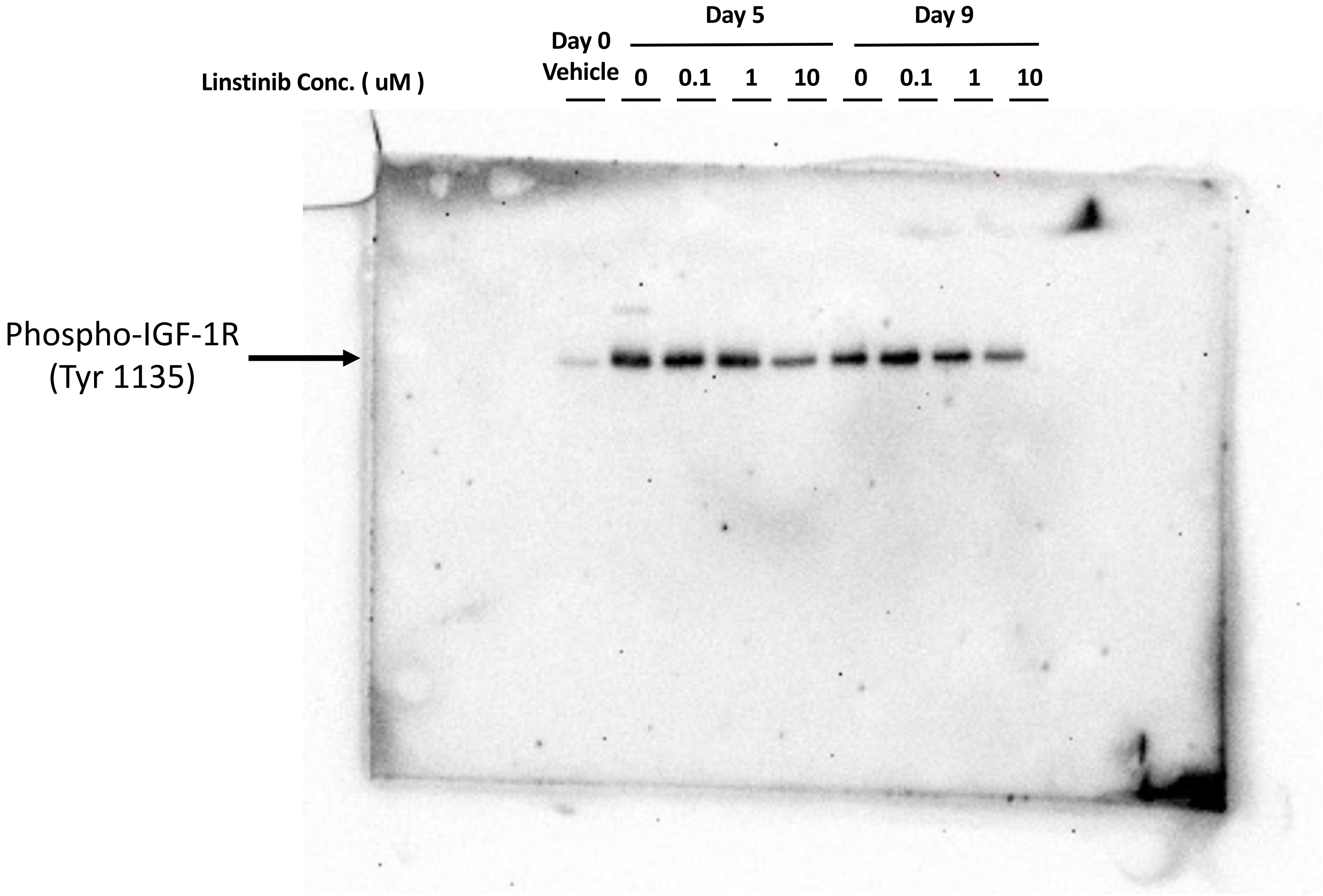
Day 5
Day 9
Day 0
Vehicle
Linstinib Conc. (uM)
0
0.1
1
10
0
0.1
1
10
Phospho-IGF-1R
(Tyr 1135)

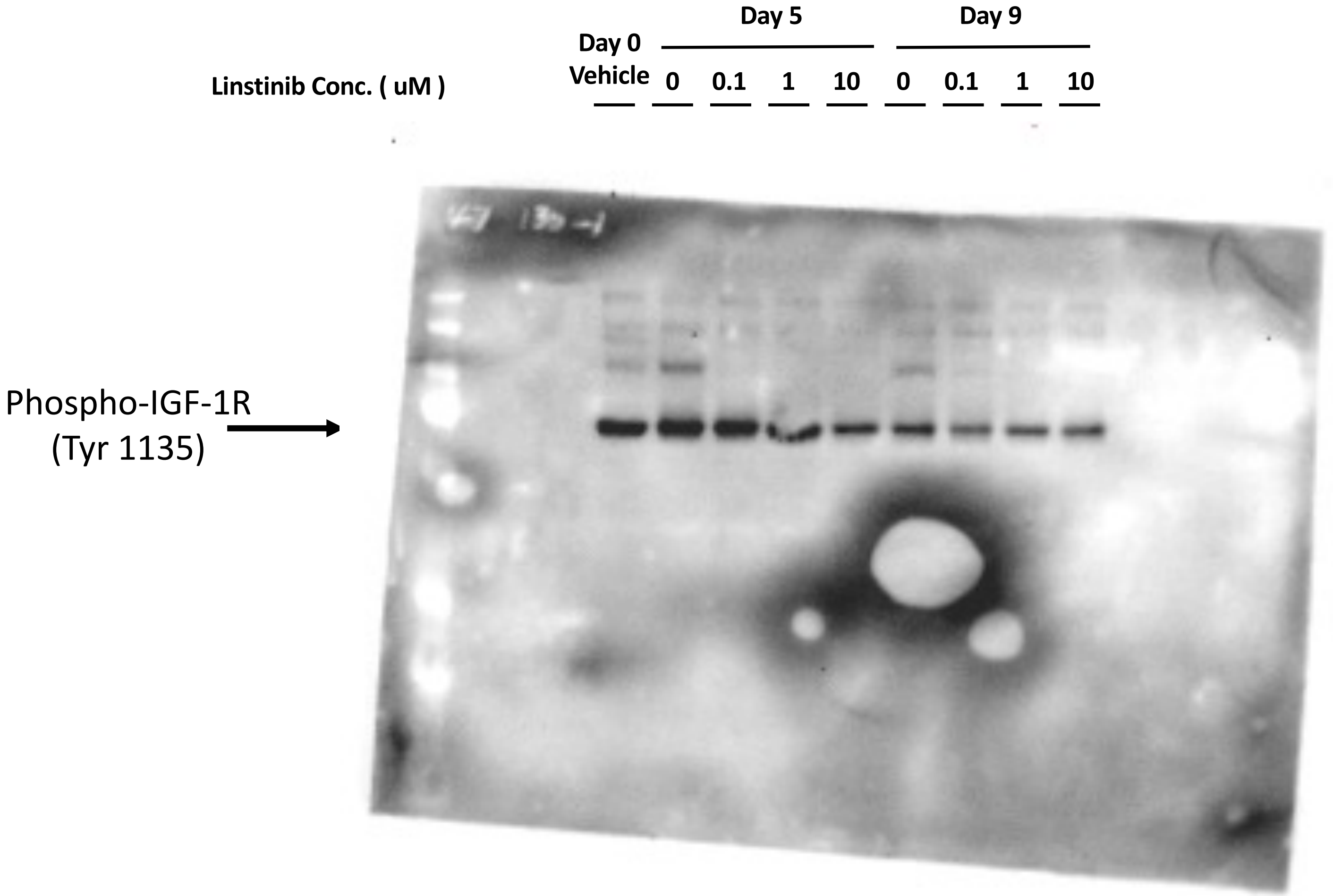
Day 5
Day 9
Day 0
Vehicle
Linstinib Conc. (uM)
0
0.1
1
10
0
0.1
1
10
Phospho-IGF-1R
(Tyr 1135)

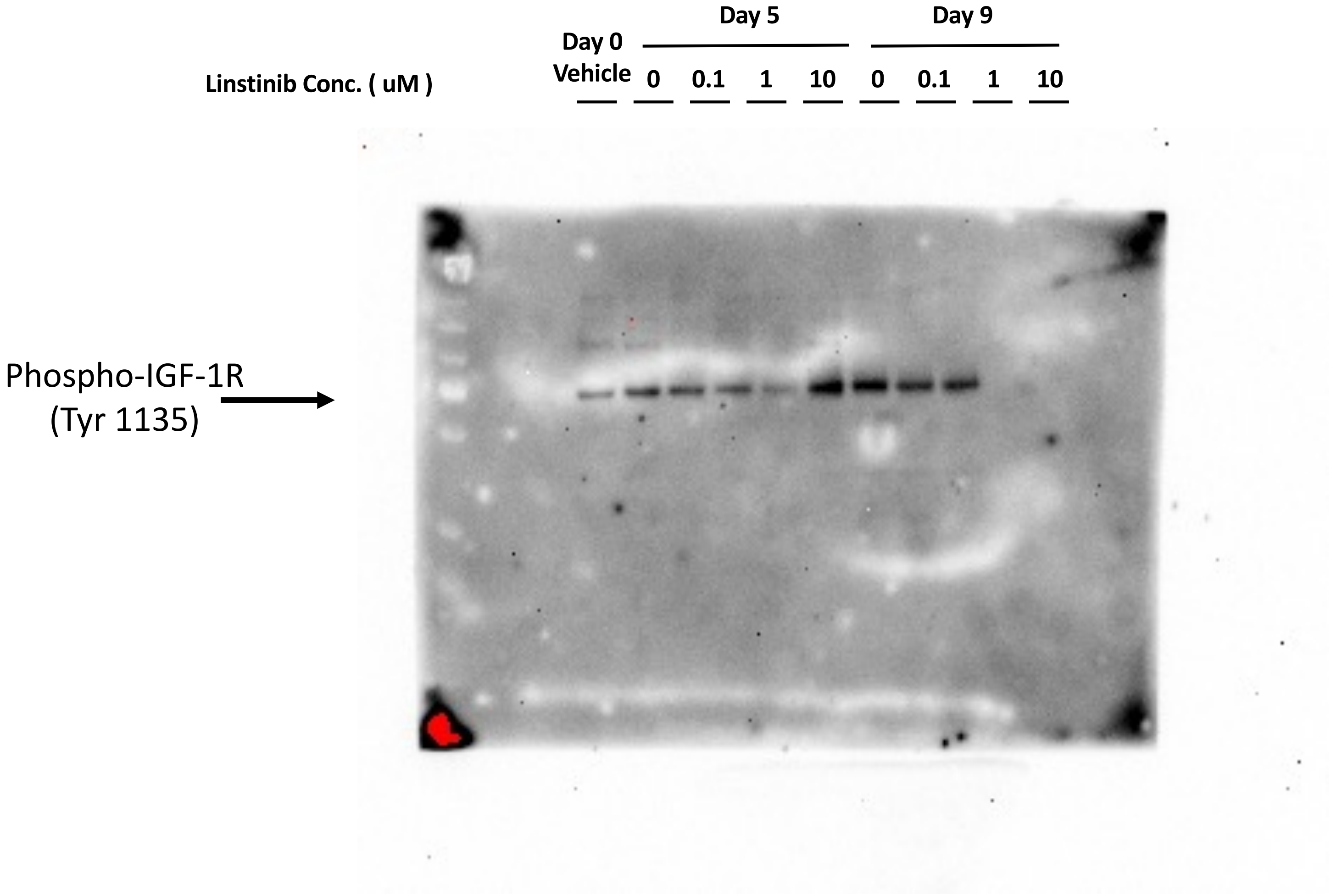
Day 5
Day 9
Day 0
Vehicle
Linstinib Conc. (uM)
0
0.1
1
10
0
0.1
1
10
Phospho-IGF-1R
(Tyr 1135)

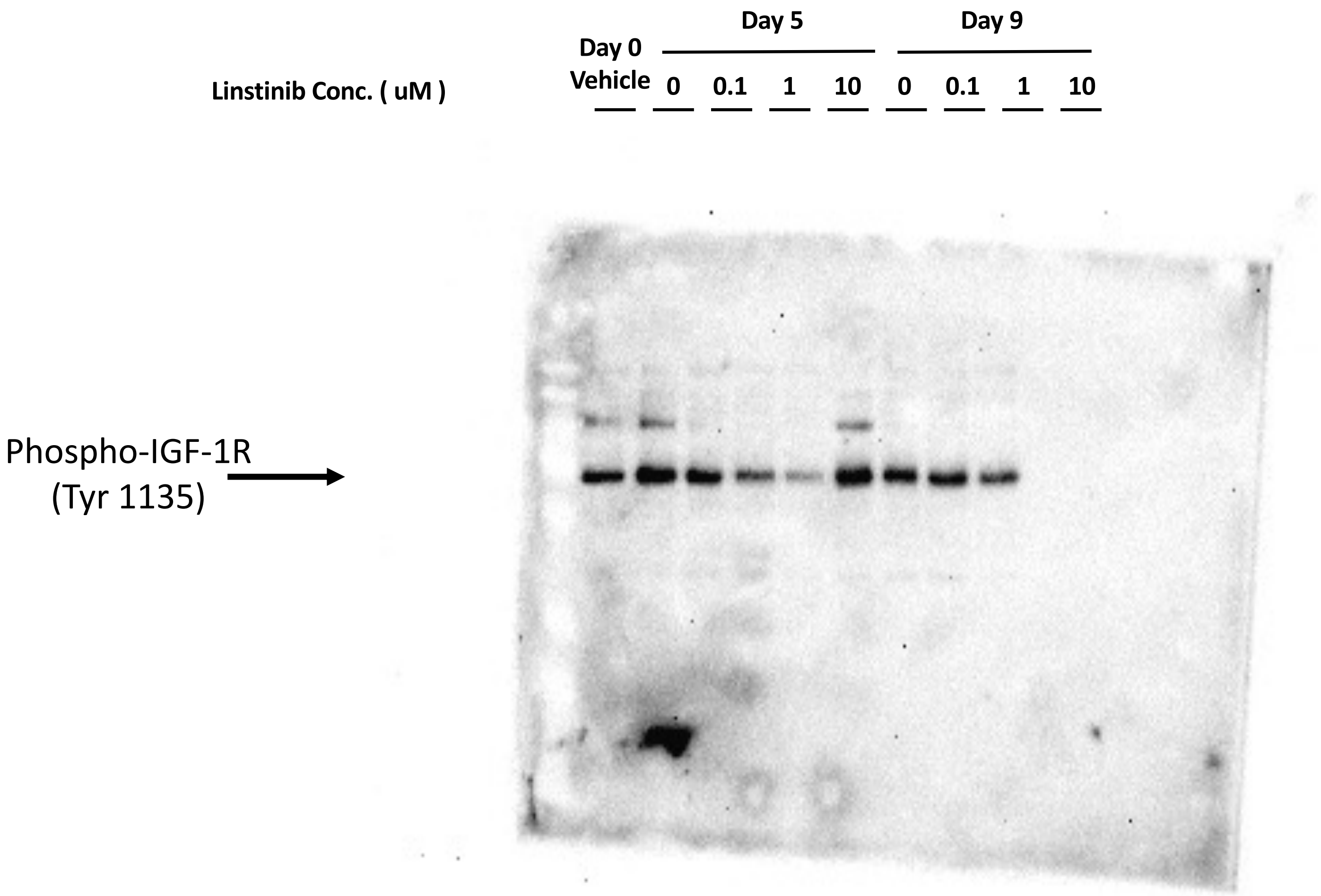
Day 5
Day 9
Day 0
Vehicle
Linstinib Conc. (uM)
0
0.1
1
10
0
0.1
1
10
Phospho-IGF-1R
(Tyr 1135)

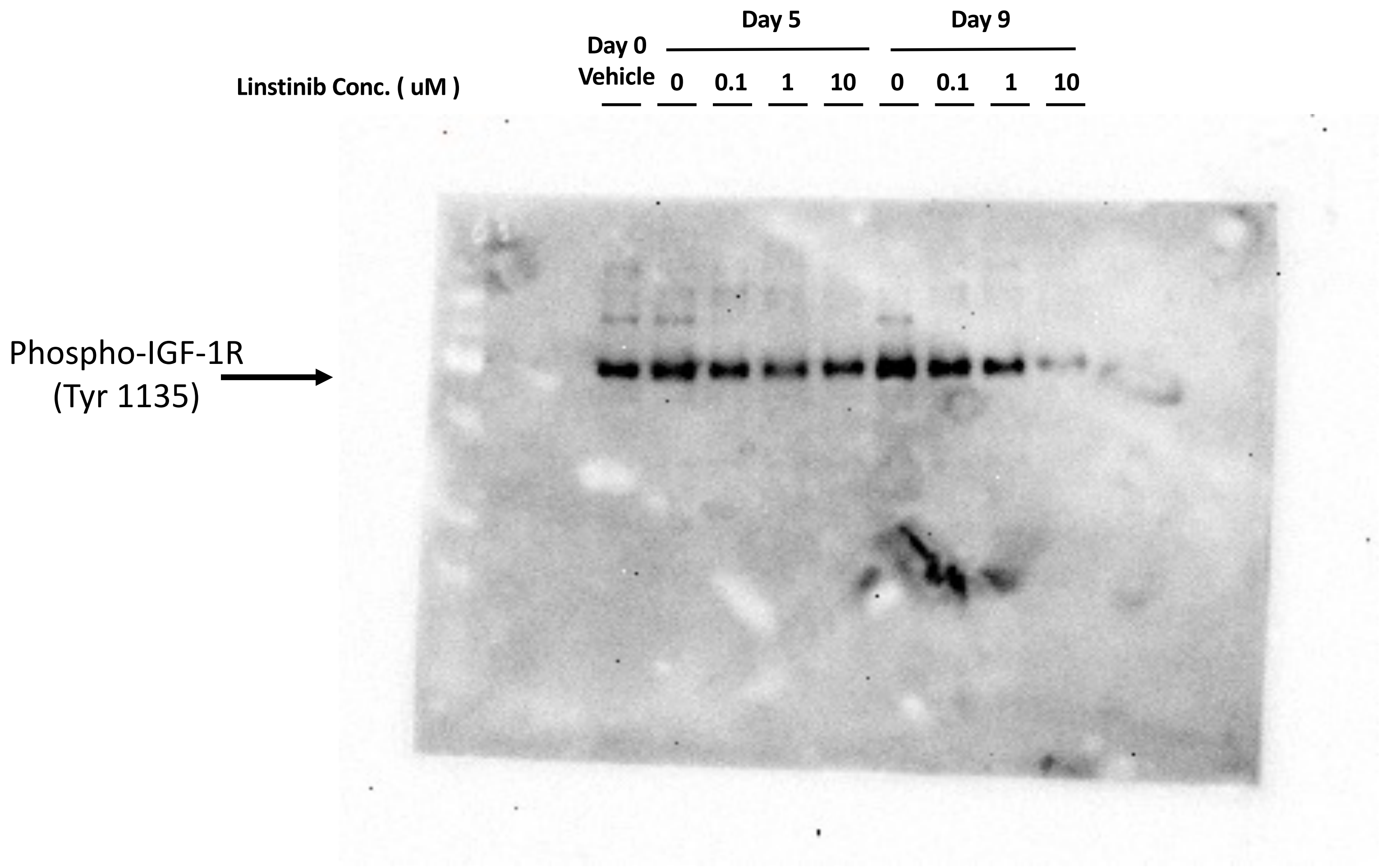

Day 5
Day 9
Day 0
Vehicle
Linstinib Conc. (uM)
0
0.1
1
10
0
0.1
1
10
Phospho-IGF-1R
(Tyr 1135)

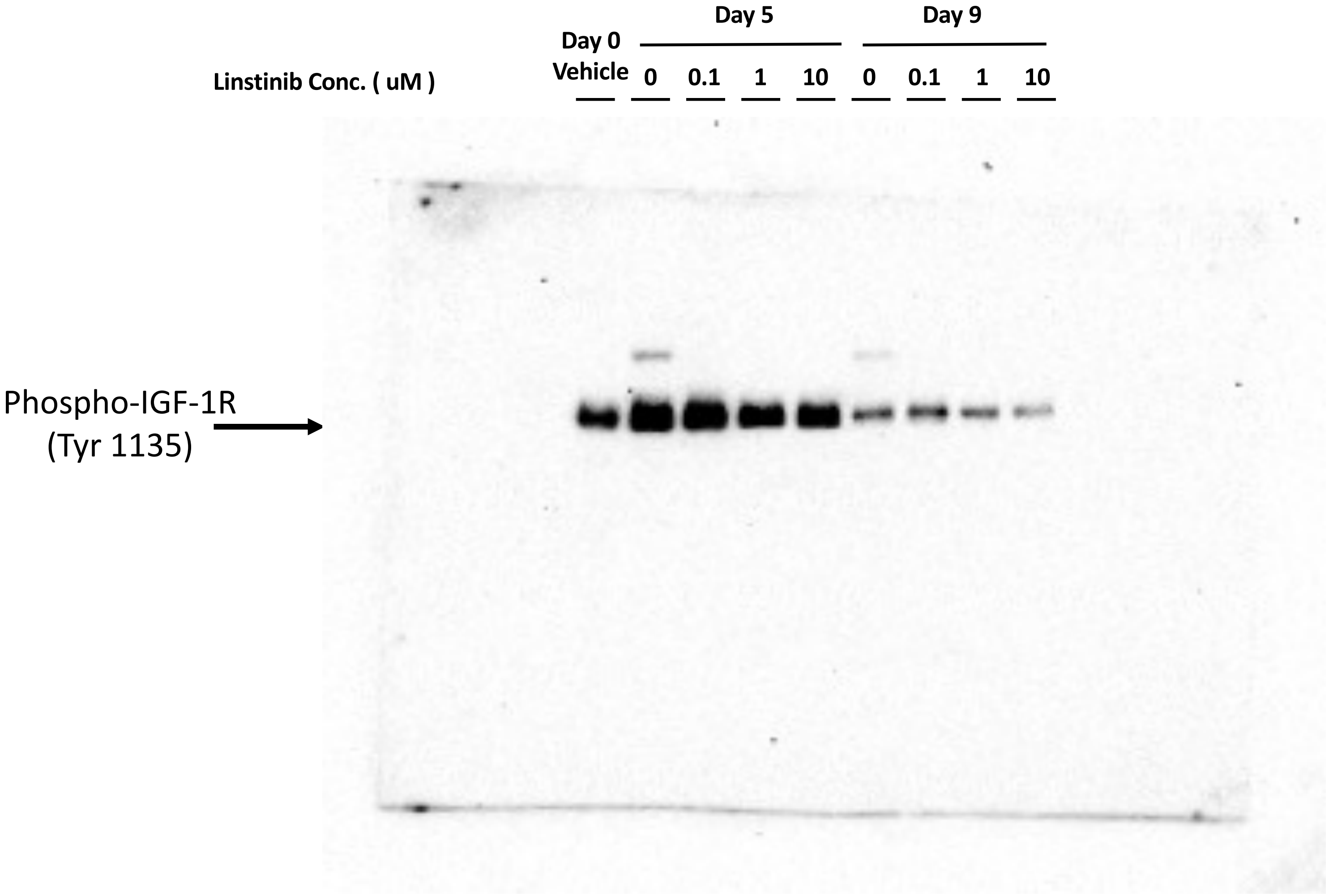
Day 5
Day 9
Day 0
Vehicle
Linstinib Conc. (uM)
0
0.1
1
10
0
0.1
1
10
Phospho-IGF-1R
(Tyr 1135)

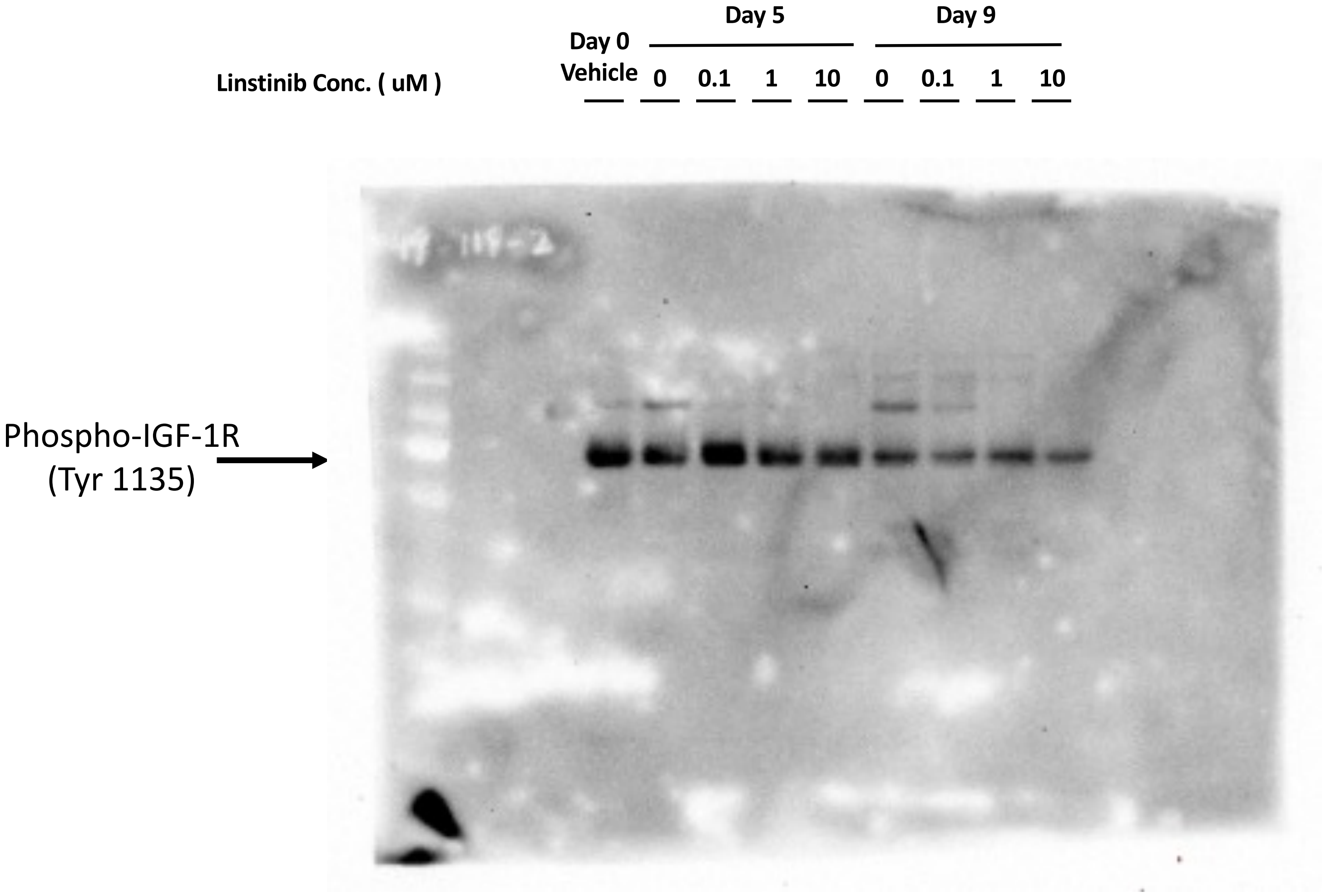
Day 5
Day 9
Day 0
Vehicle
Linstinib Conc. (uM)
0
0.1
1
10
0
0.1
1
10
Phospho-IGF-1R
(Tyr 1135)

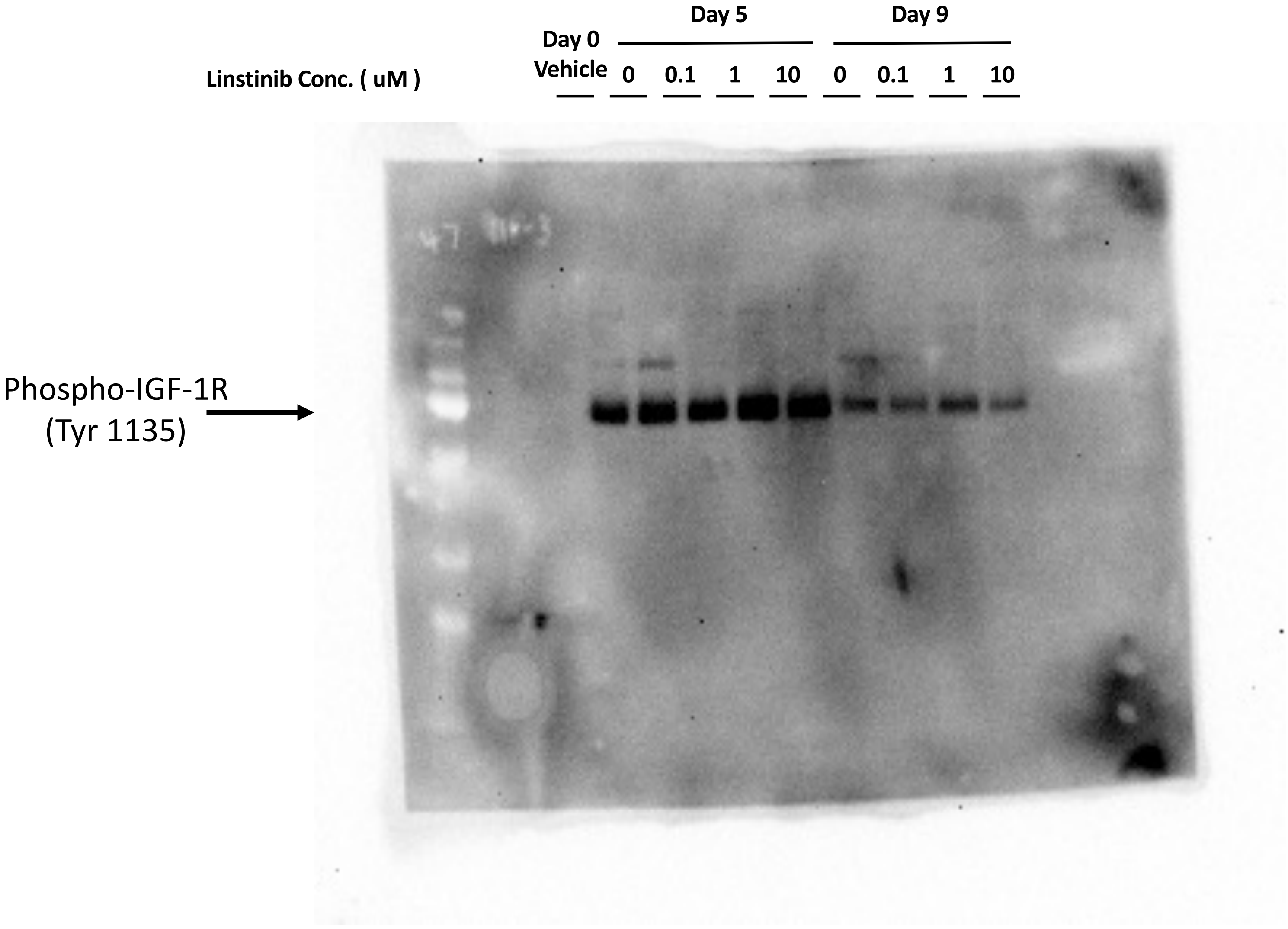
Day 5
Day 9
Day 0
Vehicle
Linstinib Conc. (uM)
0
0.1
1
10
0
0.1
1
10
Phospho-IGF-1R
(Tyr 1135)

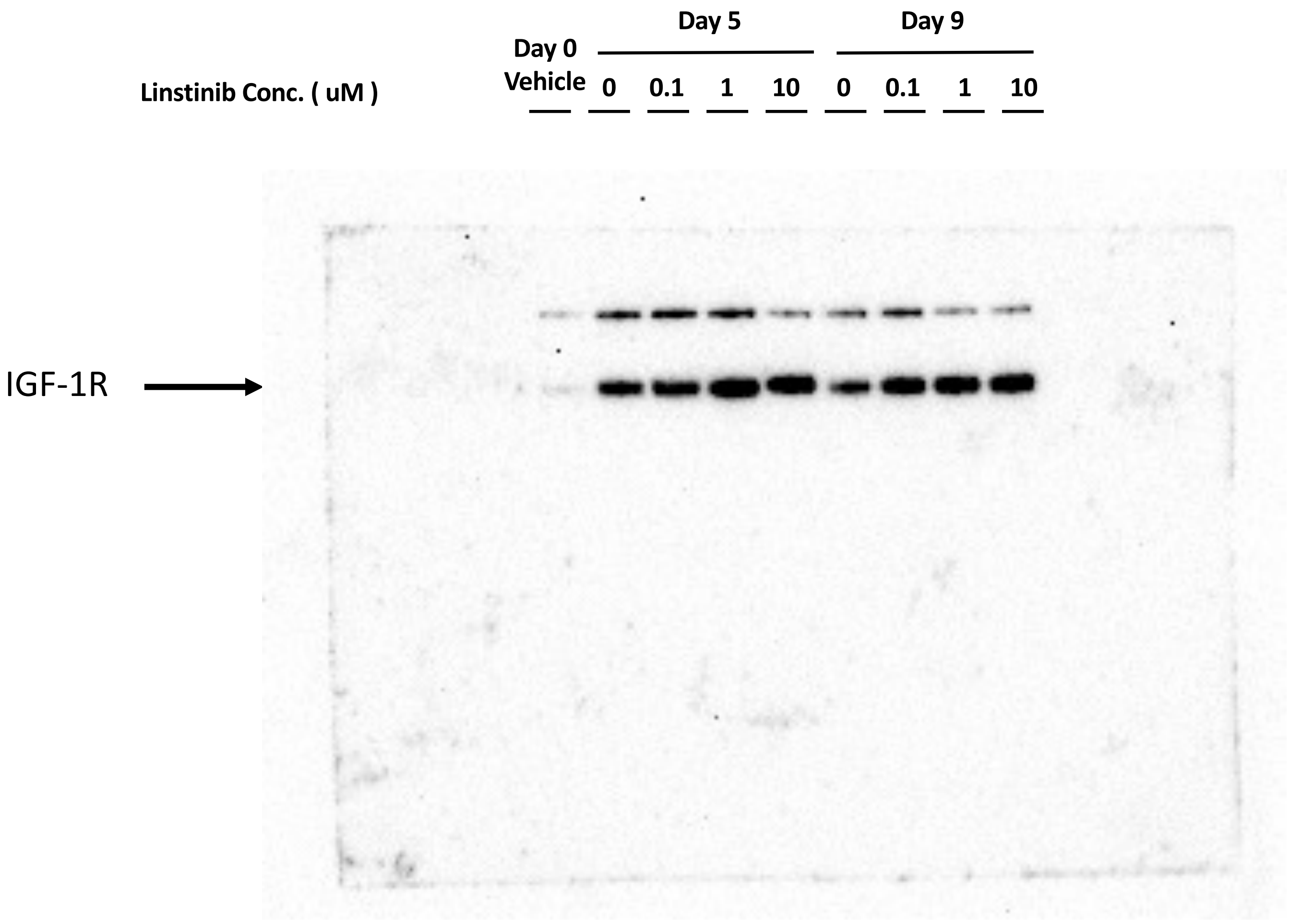
Day 5
Day 9
Day 0
Vehicle
Linstinib Conc. (uM)
0
0.1
1
10
0
0.1
1
10
IGF-1R

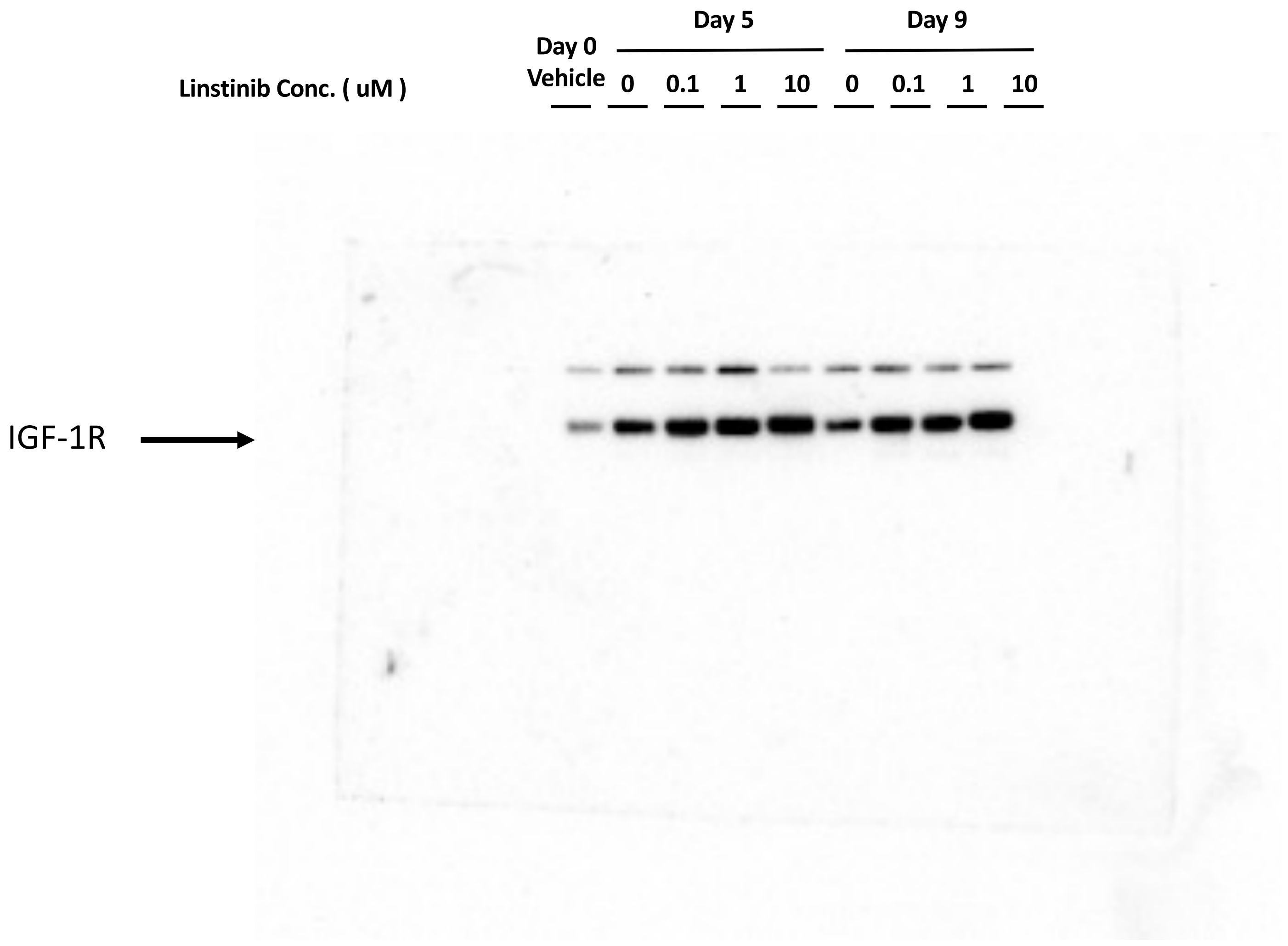

Day 5
Day 9
Day 0
Vehicle
Linstinib Conc. (uM)
0
0.1
1
10
0
0.1
1
10
IGF-1R

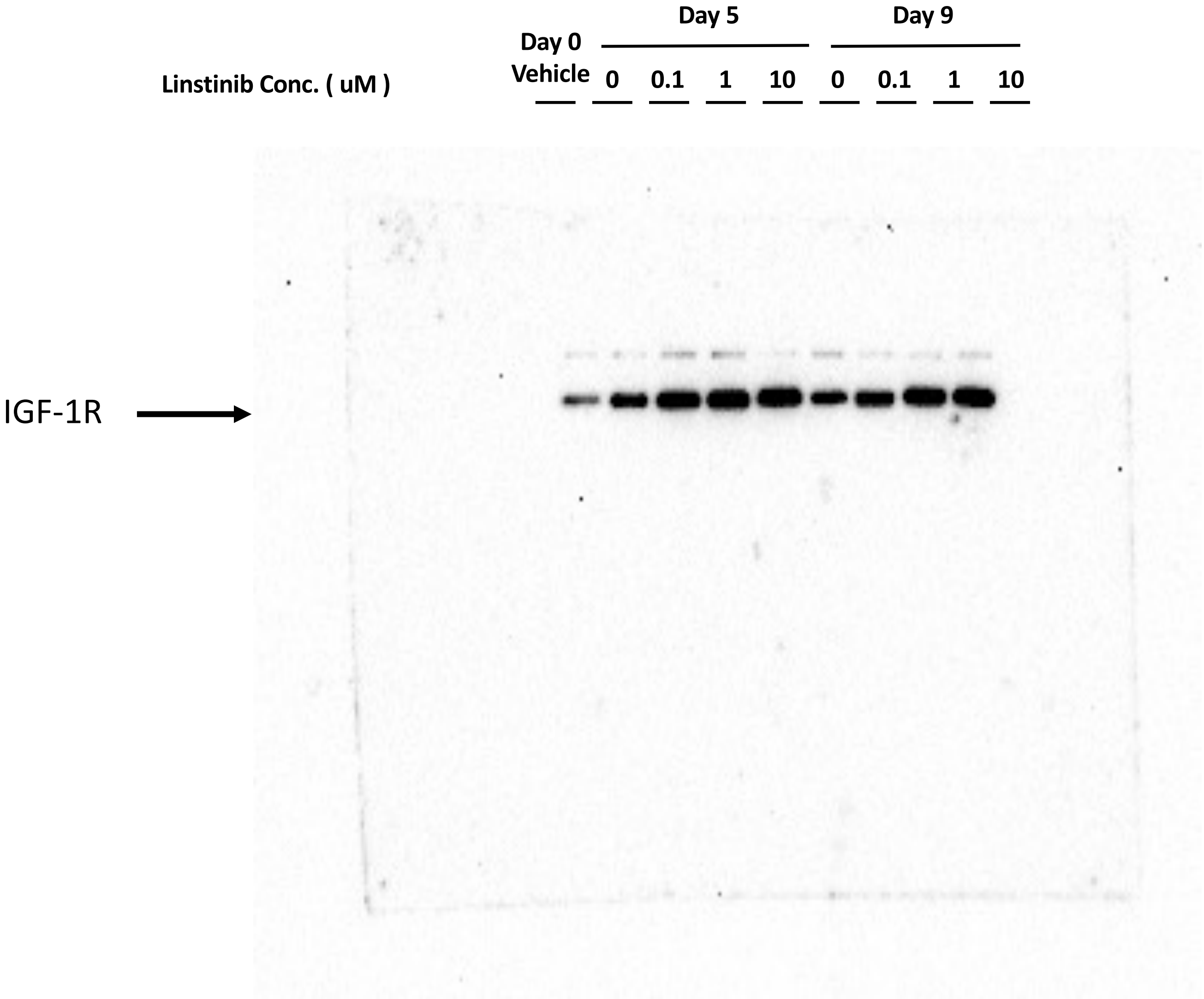
Day 5
Day 9
Day 0
Vehicle
Linstinib Conc. (uM)
0
0.1
1
10
0
0.1
1
10
IGF-1R

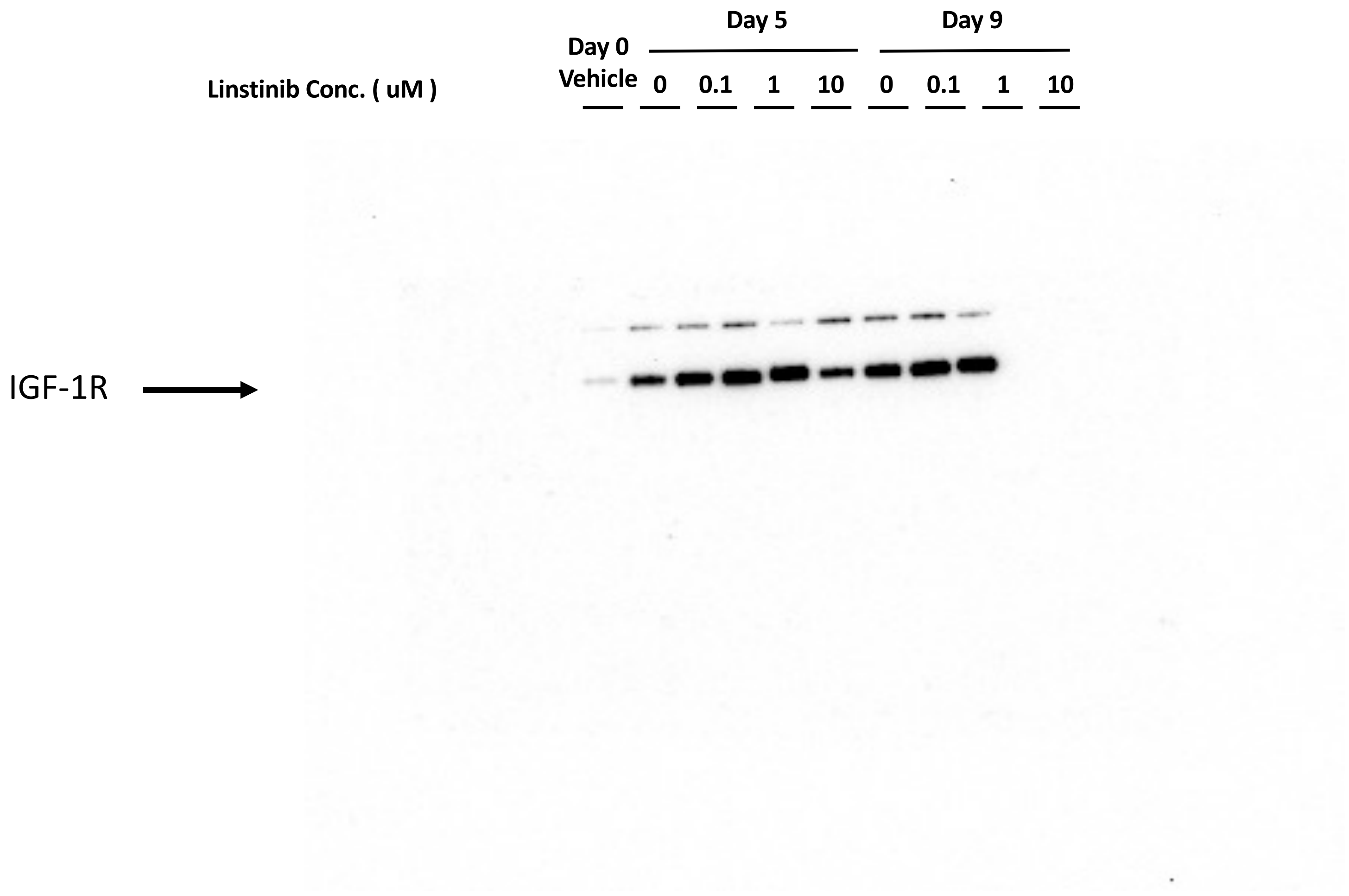
Day 5
Day 9
Day 0
Vehicle
Linstinib Conc. (uM)
0
0.1
1
10
0
0.1
1
10
IGF-1R

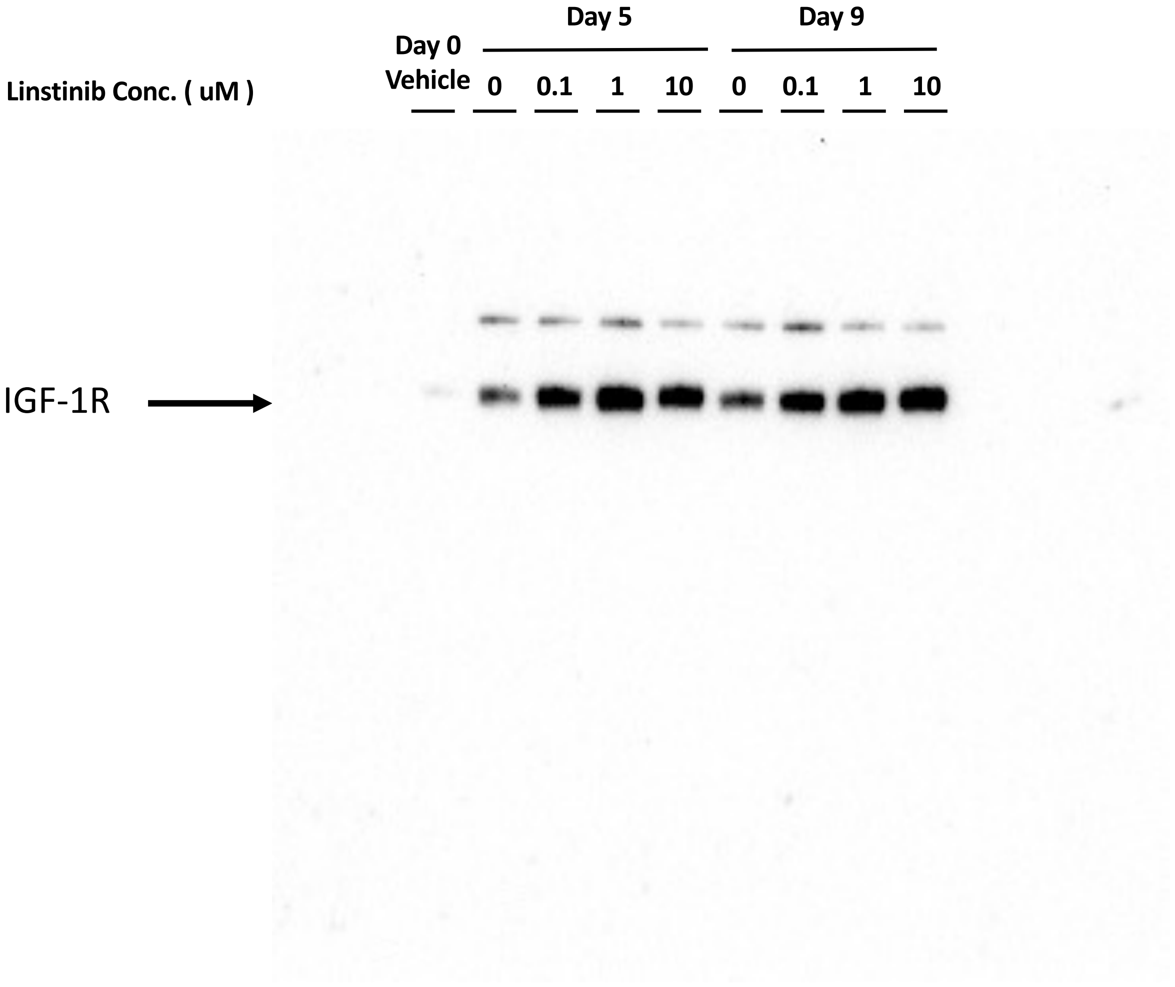
Day 5
Day 9
Day 0
Vehicle
Linstinib Conc. (uM)
0
0.1
1
10
0
0.1
1
10
IGF-1R

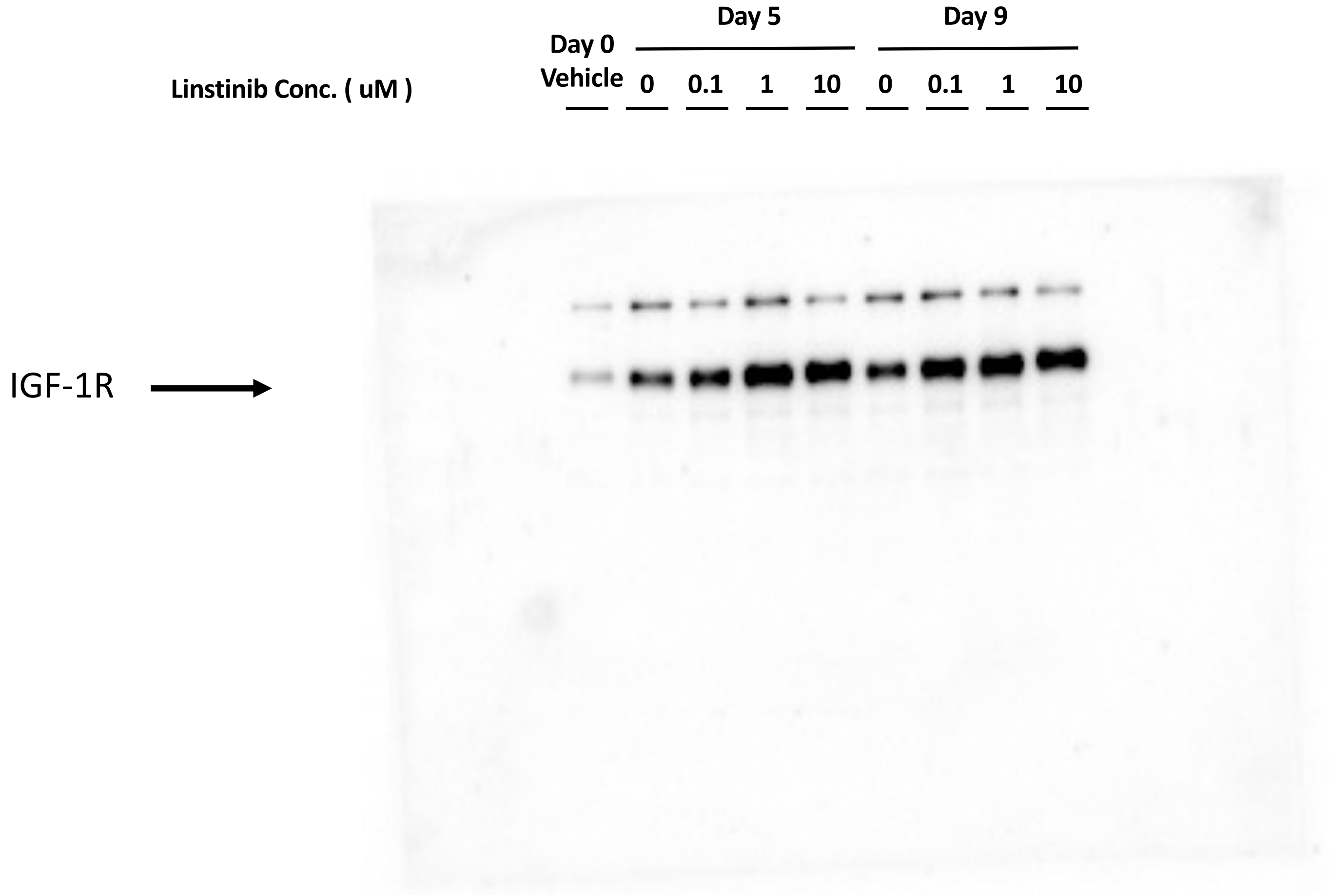

Day 0
Vehicle
Day 5
Day 9
Linstinib Conc. (uM)
0
0.1
1
10
0
0.1
1
10
IGF-1R

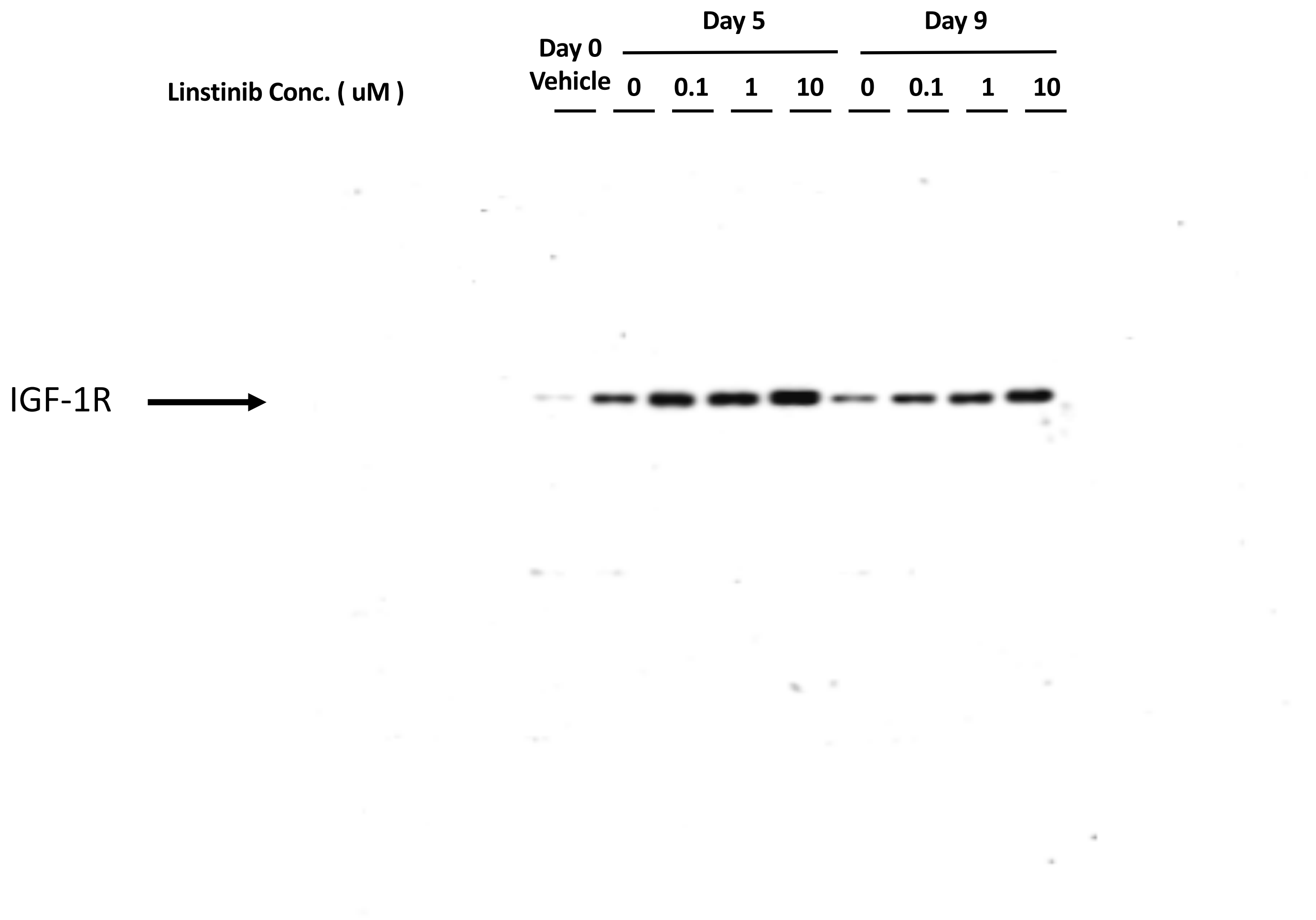
Day 5
Day 9
Day 0
Vehicle
Linstinib Conc. (uM)
0
0.1
1
10
0
0.1
1
10
IGF-1R

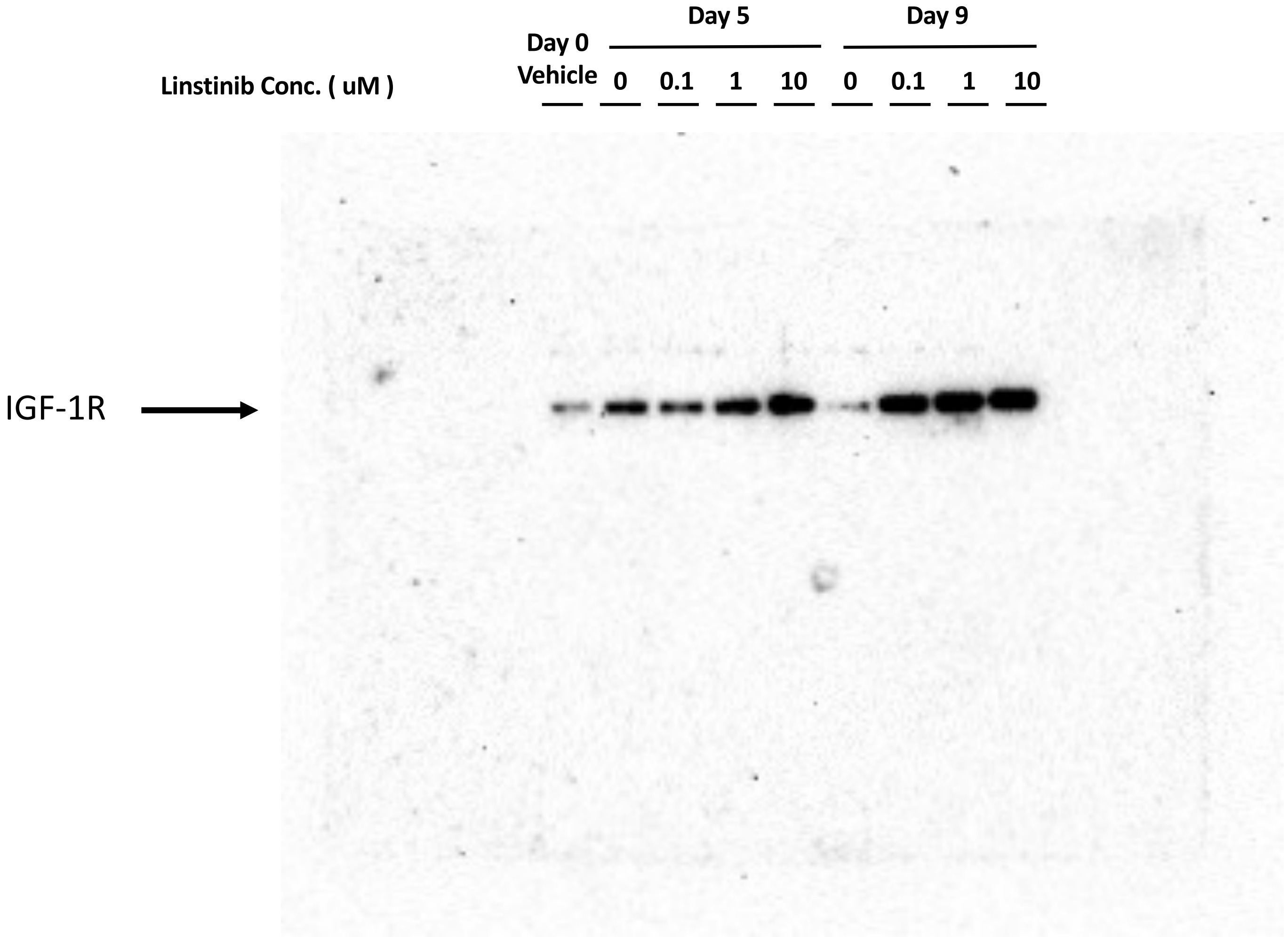
Day 5
Day 9
Day 0
Vehicle
Linstinib Conc. (uM)
0
0.1
1
10
0
0.1
1
10
IGF-1R

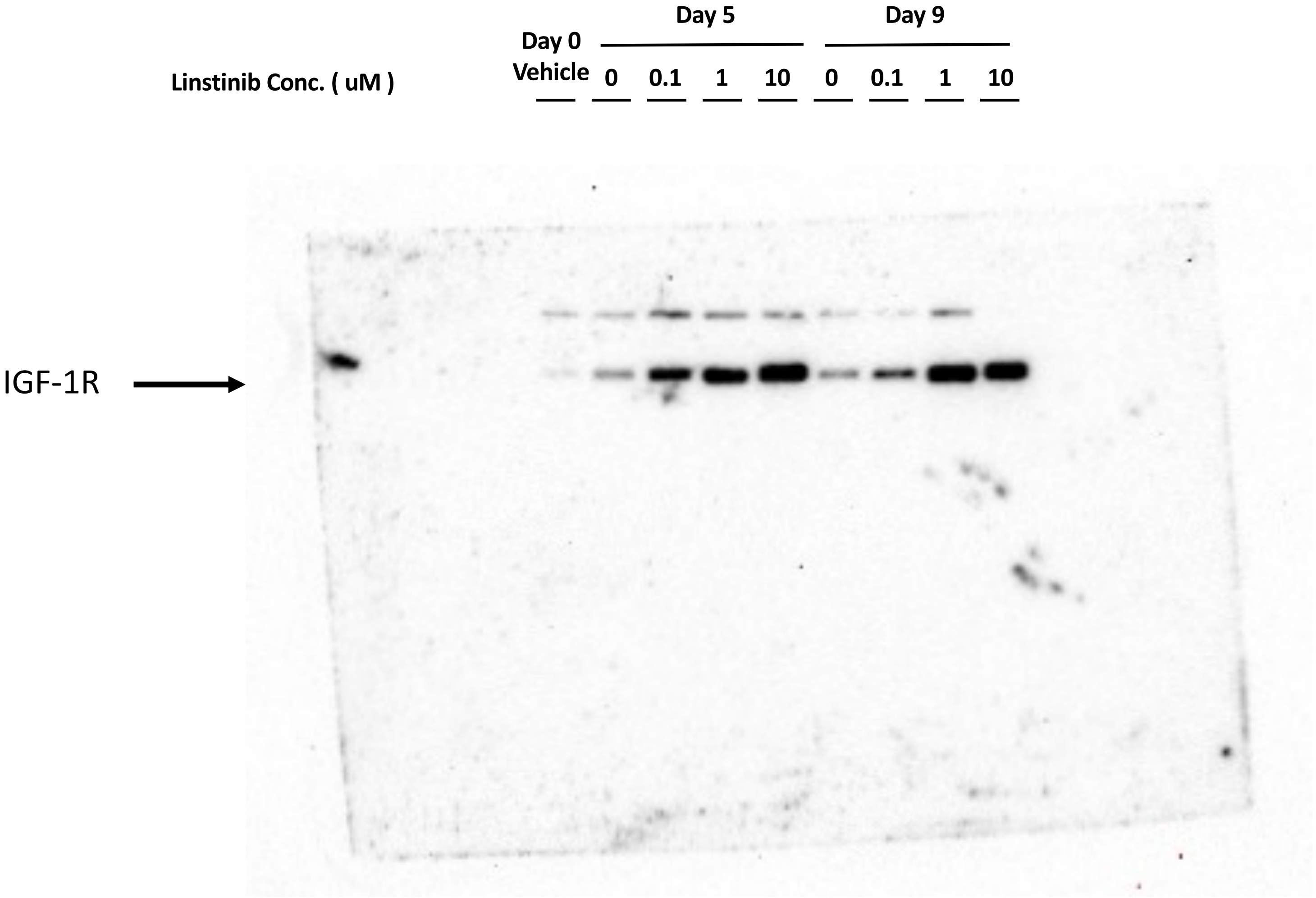
Day 5
Day 9
Day 0
Vehicle
Linstinib Conc. (uM)
0
0.1
1
10
0
0.1
1
10
IGF-1R
